# Unexpected kinetically controlled organoselenium-based isomaleimide: X-ray structure, hirshfeld surface analysis, 3D energy framework approach, and density functional theory calculation

**DOI:** 10.3389/fchem.2022.961787

**Published:** 2022-08-05

**Authors:** Saad Shaaban, Hela Ferjani, Hany M. Abd El-Lateef, Mai M. Khalaf, Mohamed Gouda, Mohamed Alaasar, Tarek A. Yousef

**Affiliations:** ^1^ Department of Chemistry, College of Science, King Faisal University, Al Hofuf, Saudi Arabia; ^2^ Department of Chemistry, Organic Chemistry Division, College of Science, Mansoura University, Mansoura, Egypt; ^3^ Department of Chemistry, College of Science, IMSIU (Imam Mohammad Ibn Saud Islamic University), Riyadh, Saudi Arabia; ^4^ Chemistry Department, Faculty of Science, Sohag University, Sohag, Egypt; ^5^ Institute of Chemistry, Martin Luther University Halle-Wittenberg, Halle (Saale), Germany; ^6^ Department of Chemistry, Faculty of Science, Cairo University, Giza, Egypt; ^7^ Toxic and Narcotic Drug, Forensic Medicine Department, Mansoura Laboratory, Medicolegal Organization, Ministry of Justice, Cairo, Egypt

**Keywords:** isomaleimide, organoselenium, crystal structure, antioxidant, hirshfeld surface analysis, DFT calculations, 3D energy framework

## Abstract

Reduction of 4,4′-diselanediyldianiline (**1**) followed by the reaction with bromo-4-(bromomethyl)benzene afforded the corresponding 4-((4-bromobenzyl)selanyl)aniline (**2**) in 85% yield. *N*-Maleanilic acid **3** was obtained in 94% yield via the reaction of selenoamine **2** with toxilic anhydride. Subsequent dehydration of *N*-maleanilic acid **3** using acetic anhydride furnished the unexpected isomaleimide 5-((4-((4-bromophenyl)selanyl)phenyl)imino)furan-2(5H)-one (**4**) instead of the maleimide **5**. The molecular structure of compound **4** was confirmed by mass spectrometry, ^1^H- and ^13^C-NMR spectroscopy, and X-ray diffraction analysis. Their cytotoxicity was assessed against two oligodendrocytes, and their respective redox properties were evaluated using 2′,7′-dichlorodihydrofluorescein diacetate (H2-DCFDA) assay. Furthermore, their antiapoptotic potential was also evaluated by flow cytometry. The compound crystallizes in triclinic P-1 space group with unit cell parameters a = 5.7880 (4) Å, b = 9.8913 (6) Å, c = 14.5951 (9) Å, V = 1731.0 (3) Å^3^ and Z = 2. The crystal packing is stabilized by intermolecular hydrogen bonding, π···π, C-Br···π stacking interactions, and other non-covalent interactions. The mapping of different Hirshfeld surfaces and 2D-fingerprint were used to investigate intermolecular interactions. The interaction energies that stabilize the crystal packing were calculated and graphically represented as framework energy diagrams. We present a computational investigation of compound 4’s molecular structure at the Density Functional Theory level using the B3LYP method and the 6-31G ++ basis set in this paper. The optimized structure matches the experimental outcome. The global reactivity descriptors and molecular electrostatic potential (M.E.P.) map emphasize the molecule’s reactive locations, allowing reactivity prediction. The charge transfer properties of molecules can be estimated by examining Frontier molecular orbitals.

## 1 Introduction

**SCHEME 1:**
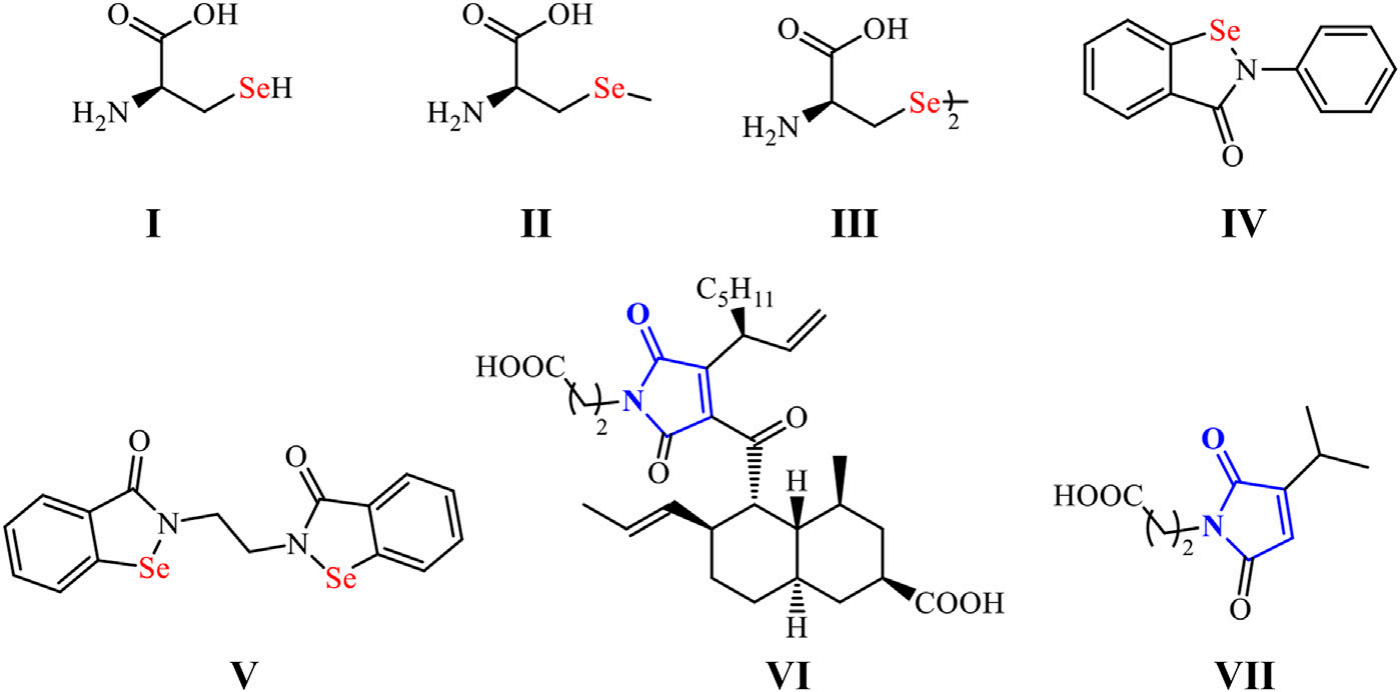
OSe compounds and naturally occurring maleimides.

Organoselenium compounds (OSe) have recently acquired significant interest as an exciting family of organic molecules with diverse applications in medicinal and organic chemistry (Phadnis, 2021; Chuai et al., 2021). They also possess enormous applications in advanced materials (Liao and Zhao, 2021). These special activities are due to the extraordinary properties of the selenium (Se) center (Handy et al., 2021). The non-metal bio-trace element Se is essential for the immune system’s normal function and protects cells from oxidative damage (Liao and Zhao, 2021; Makhal et al., 2021; Nogueira et al., 2021). It presents in most the living organisms as part of the selenoproteins and the antioxidant enzymes [e.g., thioredoxin reductases (TrxR) and glutathione peroxidase (GPx)] (Xu et al., 2020; Makhal et al., 2021; Radomska et al., 2021). Furthermore, the Se lower electronegativity (2.55), larger size (1.17 Å), and higher polarizability (3.8 Å) compared to sulfur (2.58, 1.02 Å, and 2.9 Å, respectively) made OSe compounds generally better nucleophiles (Sarma and Mugesh, 2005). Therefore, OSe compounds can react with O_2_-free radicals and thus attenuate oxidative stress-related disease progression (Rathore et al., 2019). Recently, OSe compounds have become promising candidates in cancer therapeutics (He et al., 2020). They have also manifested good histone deacetylase inhibitor activity (Adimulam et al., 2021). On the other hand, OSe compounds were extensively used as semiconductors in advanced materials, including photovoltaic cells and sodium-ion batteries and catalysts for the H_2_ evolution (Arora et al., 2021). Additionally, the pharmacological properties of OSe naturally occurring and drug molecules are attributed to the presence of the Se atom as a part of their scaffolds. Within this context, the selenocysteine (**I**), selenomethionine (**II**), and selenocystine (**III**) amino acids are present in the structure of several selenoproteins and selenoenzymes essential for the maintenance of metabolic rate, and immune responses, and oxidative homeostasis (Scheme 1) (Barbosa et al., 2017; Bartolini et al., 2017; Nogueira et al., 2021).

Furthermore, ebselen (**IV**) and ethaselen (**VI**) are among the most investigated OSe compounds with exciting GPx- and TrxR-like activities, respectively (Scheme 1) (Wang et al., 2012; Benelli et al., 2021). Indeed, they have recently entered clinical trial II as possible drugs for Meniere’s disease and non-small lung cancer, respectively (Wang et al., 2012; Shaaban et al., 2016a). We have recently directed our research toward synthesizing OSe-base maleimides (Shaaban et al., 2015a; Shaaban et al., 2015b; Shaaban et al., 2015c; Shaaban et al., 2018; Cherkaoui-Malki et al., 2019). The latter are privileged scaffolds found in many pharmacologically active drug molecules such as oxaleimide A (**VI**) and farinomalein (**VII**) (Viveki et al., 2021). Moreover, they are widely used in material science with industrial relevance, such as optoelectronic devices, resins, adhesives, rubber, and aerospace applications (Tonglairoum et al., 2016; Ravasco et al., 2019).

Toxilic anhydride reaction with amine is the common method used to prepare *N*-maleamic acids (Chen et al., 2007; Alam et al., 2022). Subsequent dehydration of the *N*-maleamic acids and ring closure affords the corresponding maleimides and isomaleimides, depending on the reaction conditions (Haval et al., 2006). Maleimides are the typical thermodynamically controlled product, whereas isomaleimides are usually kinetically controlled (Haval et al., 2006; Alam et al., 2022). Despite the numerous application and attention paid to maleimides, the synthesis of isomaleimides has gained little concern. Since the report of the 1st naphthyl-isomaleimide (Tsou et al., 1955) and new protocols are emerging describing the synthesis of such potential scaffolds; however, most of these evolving methods are limited to the dehydration of the respective maleamic acid with dehydrating agents under specified conditions. The dehydrating agents used include cyanuric chloride, oxalyl chloride, ethyl chloroformate, *N*,*N*′-dicyclohexylcarbodiimide, and 2-chloro-1,3-dimethylimidazolinium chloride, as well as triflouroacetic anhydride and propanephosphonic acid anhydride (Tsou et al., 1955; Burkitt and Gilbert, 1989; Haval et al., 2006; Guevara-Salazar et al., 2011; Haratake et al., 2011; Tonglairoum et al., 2016; Gao et al., 2019; Ravasco et al., 2019; Alam et al., 2022). Unfortunately, these protocols are limited by the lack of dehydrating agent with wide substrate scope and harsh conditions and long reaction time as well as the poor selectivity often associated with the formation of the undesired maleimide side product. Therefore, the development of an alternative, efficient, and general synthetic methods of isomaleimides remains a challenge.

We herein report the accidental synthesis and crystal structure of isomaleimide **4**. Its cytoprotective activity was evaluated against oligodendrocytes. Furthermore, its antioxidant and antiapoptotic actives were also assessed. The Hirshfeld surface analysis description was used to characterize the nature of intermolecular interactions in crystal packing. Moreover, DFT simulations were used to optimize compound 4’s structure in its isolated condition. Furthermore, global reactivity descriptors and complementary interaction sites in isomaleimide **4** were identified using Frontier molecular orbitals (F.M.O.) and M.E.P. mapping studies.

## 2 Materials and methods

### 2.1 Synthesis of isomaleimide **4**

**SCHEME 2:**
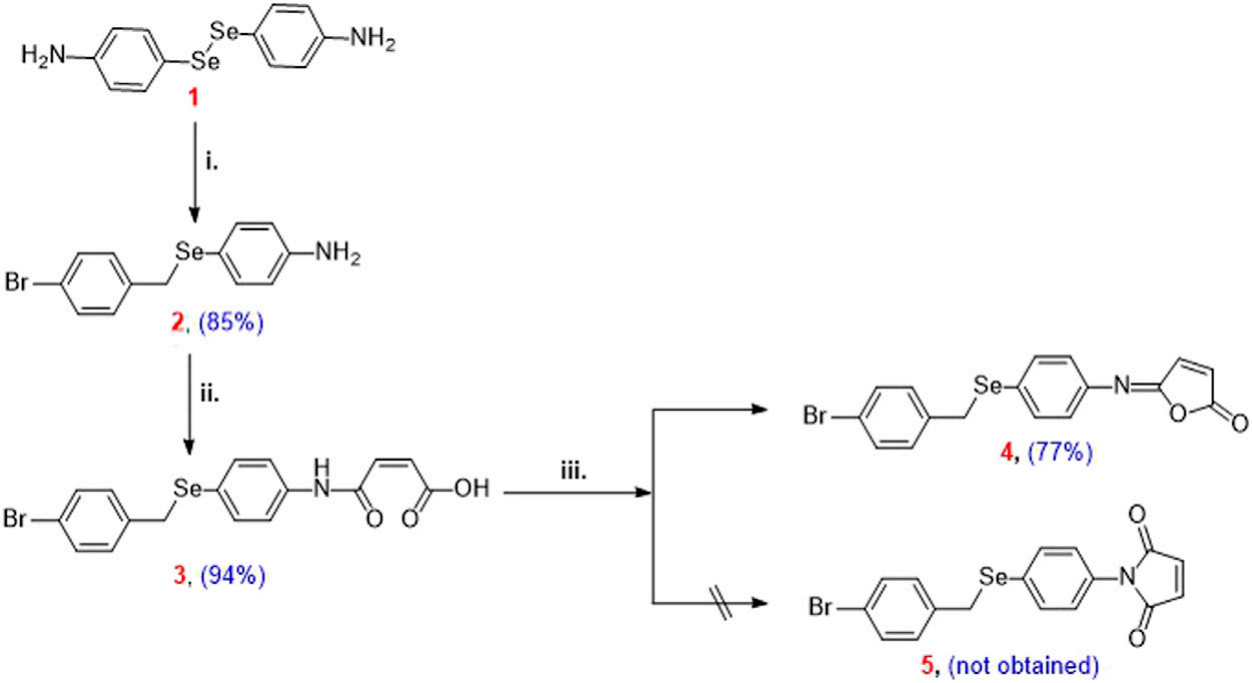
Reagents and conditions: (i) 4,4′-diselanediyldianiline (**1**) (2.5 mmol), bromo-4-(bromomethyl)benzene (3 mmol), EtOH (30 ml), NaBH_4_ (12.5 mmol); (ii) 4-((4-bromobenzyl)selanyl)aniline (**2**) (2.5 mmol), toluene (15 ml), toxilic anhydride (2.5 mmol), 4 h, r.t.; (iii) *N*-maleanilic acid **3** (2.5 mmol), Ac_2_O (8 ml), 250 mg NaOAc, 3 h, 40–50°C.

Isomaleimide **4** was synthesized in good yield (77%) over three steps by the reduction of the diselenide **1** using NaBH_4_ and subsequent reaction with bromo-4-(bromomethyl)benzene followed by reaction with toxilic anhydride and subsequent ring closure using acetic anhydride and sodium acetate (Scheme 2) (Shaaban et al., 2018). Diselenide 1 was synthesized starting from aniline according to our reported method (Shaaban et al., 2018).

#### 2.1.1 Synthesis of 4-((4-bromobenzyl)selanyl)aniline (2)

Compound **2** was synthesized from the reaction of diselenide **1** (344 mg, 1 mmol), 4-bromobenzyl bromide (550 mg, 2.2 mmol), aliquat 336 (45 mg, 5% mol) and sodium tetrahydridoborate (189.15 mg, 5 mmol) in ethylactetae and water mixed solvent (40 ml, 1:1) under reflux for 3 h. The formation of compound **2** was followed by TLC (petroleum ether: EtOAc = 6:1). The solvent was evaporated and the product was purified by column chromatography (petroleum ether: EtOAc = 6:1.5) as white solid; Yield: 310.31 mg (91%); mp 156–158°C (see supporting information for the analytical details).

#### 2.1.2 Synthesis of 4-((4-((4-bromobenzyl)selanyl)phenyl)amino)-4-oxobut-2-enoic acid (3)

Compound **3** was synthesized from the reaction of compound **2** (344 mg, 1 mmol) and maleic anhydride (98 mg, 1 mmol) in dry toluene (5 ml) with stirring at r.t. for 3 h. The formation of compound **3** was monitored by TLC (chloroform: methanol = 8:1). The product was purified by column chromatography (chloroform: methanol = 6:1) to give yellow solid; Yield: 412.66 mg (94%); mp 215–217°C (see supporting information for the analytical details).

#### 2.1.3 Synthesis of 4-((4-((4-Bromobenzyl)selanyl)phenyl)imino)-1H-pyrrol-2(5H)-one (4)

Compound **4** was synthesized from the gentle heating of compound **3** (439 mg, 1 mmol), acetic anhydride (3 ml), and sodium acetate (100 mg) for 2 h at 50–60°C. Water was added and the resulting mixture was extracted with CH2Cl2 (200 ml), dried with Mg2SO4, CH2Cl2 was evaporated and the residue was purified by silica gel chromatography. The progress of the product formation was followed by TLC petroleum ether: EtOAc = 8:1, Rf = 0.45, purified by column silica gel chromatography with petroleum ether: EtOAc = 6:1. Yellow solid; Yield: 324.17 mg (77%), mp 185–187°C. 1H NMR (300 MHz, CDCl3) *δ* 7.47–7.29 (m, 4H, Ar-H), 7.25–7.14 (m, 2H, Ar-H), 7.04–6.96 (m, 2H, Ar-H), 6.76 (d, J = 5.5 Hz, 1H, CH=), 6.62 (d, J = 5.5 Hz, 1H, CH=), 4.01 (s, 2H, SeCH2); 13C NMR (75 MHz, CDCl3) *δ* 166.94, 150.34, 143.16, 142.77, 137.50, 134.29, 134.02, 131.59, 130.49, 127.93, 125.94, 120.83, 31.52; MS (ESI): *m/z* = found 478.92 [M^+^ + CH_3_COO^−^]; calcd. 420.92 [M^+^]; HRMS calcd. for C_17_H_12_BrNO_2_Se [M^+^ + 1]: 475.93688, found 477.93289 [M^+^ + CH_3_COO^−^].

### 2.2 Biological evaluation

The antiproliferative activities of OSe compounds **2**, **3**, and **4** were assessed against two oligodendrocyte cell lines, namely, 158 N and 158 JP, by the M.T.T. assay using 7-ketocholesterol as reference control (Supporting info, Supplementary Table S1) (Vejux et al., 2005; Nury et al., 2014). The redox profile of the compounds was evaluated using the H2-DCFDA assay, and their antiapoptotic potential was also assessed by flow cytometry (Wilhelm et al., 2009; Karlsson et al., 2010; Shaaban et al., 2018).

### 2.3 Crystal structure measurement

A suitable crystal of compound **4** was mounted on a glass fiber loop. At 100 K, X-ray data were collected using a Bruker D8 VENTURE diffractometer and graphite-monochromated Mo(Kα) radiation (*λ* = 0.71073 Å). The full data set was used to calculate and refine unit cell parameters. S.A.D.A.B.S. Krause et al. (2015) was used to scale reflections and apply absorption corrections. The structure was solved using the ShelXT (Wilhelm et al., 2009; Karlsson et al., 2010) structure solution program using Direct Methods and refined using the ShelXL (Wilhelm et al., 2009) refinement package using Least Squares minimization using Olex2 (Krause et al., 2015). Hydrogen atoms were placed in calculated positions (C-H = 0.95–0.99 Å) and were constrained to ride on their parent atoms, with U_iso_(H) = 1.2U_eq_(C). The crystal packing and molecular packing were drawn using the program Diamond 3 (Sheldrick, 2008) and MERCURY (Macrae et al., 2020). The details of structural refinement and crystal data are reported in Table 1. Crystallographic data from the structural analysis have been deposited with the Cambridge Crystallographic Data Center, Nos CCDC-1472956 Copies of this information may be obtained free of charge from: The Director, C.C.D.C., 12 Union Road, Cambridge CB2 1EZ, United Kingdom. Fax: +44(1223)336-033, e-mail: deposit@ccdc.cam.ac.uk, or http://www.ccdc.cam.ac.uk.

**TABLE 1:** Crystal data and structure refinement for compound **4**.

Empirical formula	C_17_H_12_NO_2_SeBr
M_r_	421.15
Temperature/K	99.99
Crystal system	Triclinic
*Space group*	*P-1*
*a (Å)*	5.7880 (4)
*b (Å)*	9.8913 (6)
*c (Å)*	14.5951 (9)
*α* (°)	102.785 (2)
*β* (°)	100.935 (2)
*γ* (°)	103.971 (2)
Volume (Å^3^)	764.22 (9)
Z	2
ρ_calc_ (g/cm^3^)	1.830
μ (mm^−1^)	5.076
F (000)	412.0
Crystal size (mm^3^)	0.37 × 0.37 × 0.12
*θ* min/*θ* max (°)	5.972–55.534
Index ranges	−7 ≤ h ≤ 7, −12 ≤ k ≤ 12, −19 ≤ l ≤ 19
Reflections collected	32,442
Independent reflections	3,565
Data/restraints/parameters	3,565/0/199
Goodness-of-fit on F^2^ = GOOF	1.132
R1 [F^2^ > 2 σ (F^2^)]	0.0254
wR_2_ (F^2^)	0.0527
ρ_max_/ρ_min_ (e Å^−3^)	0.70/−0.62

a, b and c are the unit cell parameters

### 2.4 Hirshfeld surface, 2D-fingerprint plot, and interaction energy calculations

Hirshfeld surfaces (Spackman and Jayatilaka, 2009) were mapped with property d_norm_, and 2D-fingerprint plots were created with CrystalExplorer 17 (Spackman and Jayatilaka, 2009). Hirshfeld surfaces and 2D-fingerprint plots are excellent visualization tools for comparing intermolecular interactions in constructing various supramolecular motifs in the crystal structure. The 2D-fingerprint plot decomposes Hirshfeld surfaces into the contributions of different intermolecular interactions found in the crystal structure. The red, white, and blue colors on the Hirshfeld surface indicate shorter, equal, and longer contacts than the sum of the van der Waals radii, respectively. Furthermore, the crystal structure was analyzed using TONTO (Spackman and Jayatilaka, 2009; Spackman et al., 2021)**,** with the CE-HF···HF/3-21G energy model (Sloot et al., 2003), starting with the .cif files derived from single-crystal X-ray diffraction data. An energy framework is a one-of-a-kind tool for visualizing crystal structures’ supramolecular architecture. To allow for comparison, total energy (E_tot_) is divided into electrostatic (E_ele_), polarization (E_pol_), dispersion (E_dis_), and repulsion (E_rep_) contribution energies, with cylinders representing the relative strength of the molecular packing and fixed at a scale factor of 200 and cutoff energy of 5 kJ/mol.

### 2.5 Theoretical calculations

The Gaussian 09 software (Shaaban et al., 2016b) was used for all calculations. The B3LYP functional (Becke’s three-parameter nonlocal exchange function with the Lee-Yang-Parr correlation function) (Hughes and Appel, 2019; Shaaban et al., 2021) was employed with the Density Functional Theory (DFT) approach. The geometrical optimizations were carried out in the gas phase and at the minima, and frequency calculations were used to confirm them; they accorded well with the experimental structure data. As a result, it was possible to compare energy and other physicochemical parameters with confidence. Chemical potential (*μ*), electronegativity (*χ*), electrophilicity index (*ω*) and chemical hardness (*η*), and softness (S) were all calculated using the corresponding HOMO and LUMO energies (Allen, 2002; Shaaban et al., 2019).

## 3 Results and discussion

### 3.1 Design and synthesis of isomaleimide **4**

4,4′-Diselanediyldianiline (**1**) was used as the starting building block to prepare the target isomaleimide **4** (Shaaban et al., 2016b). In this regard, reduction of **1** with NaBH_4_ furnished the respective sodium phenylselenolate, which in turn trapped in ethanol and reacted with bromo-4-(bromomethyl)benzene to give Ose amine **2** in 85% yield (Scheme 2). The reaction of Ose amine 2 with toxilic anhydride in toluene at room temperature furnished the respective *N*-maleanilic acid 3 in excellent yield (94%). Surprisingly, gentle heating of compound **3** with Ac_2_O in the presence of NaOAc afforded the unexpected kinetically controlled isomaleimide **4** instead of thermodynamically controlled 1-(4-((4-bromobenzyl)selanyl)phenyl)-1H-pyrrole-2,5-dione (5) (Scheme 2) (Shaaban et al., 2018).

### 3.2 Charachterization of compound isomaleimide **4**

The structure of isomaleimide **4** was established by ^1^HNMR and ^13^CNMR spectroscopy. ^1^HNMR of isomaleimide **4** showed the characteristic upfield singlet signal of the methylene fragment (CH_2_) at *δ* 4.01 ppm with a coupling constant (*J*) at 5.5 Hz. The characteristic isomaleimide vinylic protons appeared at *δ* 4.01 ppm *δ* 6.76 and 6.62 ppm. The rest eight aromatic protons appeared as multiplet signals at *δ* 7.47, 7.25, and 7.04 ppm. Furthermore, the isomaleimide **4** showed 13 carbon signals in the ^13^CNMR spectroscopy. The aliphatic methylene carbon appeared upfield at *δ* 31.52 ppm. On the other hand, one carbonyl signal appeared downfield at *δ* 166.94 ppm and the azomethine (N=C) at *δ* 150.34 ppm. The vinylic carbons appeared at *δ* 137.50 and *δ* 127.93 ppm. The rest eight aromatic carbons appeared at *δ* 143.16–120.83 ppm.

### 3.3 Biology

Oligodendrocytes are susceptible to deterioration by oxygen and nitrogen reactive species (R.O.S. and R.N.S.), affecting the transmission of the neuronal signal and the proper axon function (Shaaban et al., 2018; Hughes and Appel, 2019; Shaaban et al., 2021). OSe compounds have recently manifested potential chemoprotective and antioxidant properties (Shaaban et al., 2015a; Shaaban et al., 2016b; Shaaban et al., 2019). The cytoprotective properties of OSe compounds **2**, **3**, and **4** were assessed in 158N and 158 JP cells. Interestingly, OSe compounds **2** and **4** did not show any apparent toxicity (IC_50_ ≥ 100 µM), whereas the *N*-maleanilic acid **3** showed moderate-low cytotoxicity (Supporting info, Supplementary Table S1). These results encourage the further assessment of their redox properties. Considering that OSe compounds are good nucleophilic reductants, they can react with R.O.S. and R.N.S. to protect tissues from oxidative damage (OD) (Sarma and Mugesh, 2005; Chuai et al., 2021; Nogueira et al., 2021). Oligodendrocytes are usually vulnerable to OD; thus, our main objective is further to estimate the antioxidant potential of compounds **2**, **3**, and **4** using the H2-DCFDA (Supporting info, Supplementary Table S1). Interestingly, all compounds significantly diminished the R.O.S. levels in 158N and were even more significant than vitamin E.

Furthermore, the antiapoptotic activities of compounds **2**, **3**, and **4** were also evaluated by flow cytometry in 158N cells stained with P.I. using 7Kc as apoptosis stimulator and the positive reference (Supporting info, Supplementary Figure S1). Among the tested compounds, the *N*-maleanilic acid **3** lowered, in a concentration-dependent manner, the formation of SubG1 peak. To this point, *N*-maleanilic acid **3** showed antioxidant and antiapoptotic activities probably via diminishing the R.O.S. levels. The *N*-maleanilic acid **3** amphiphilic characters favor its crossing through the cell membrane and, therefore, its better activity.

### 3.4 Analysis of the molecular packing

**FIGURE 1:**
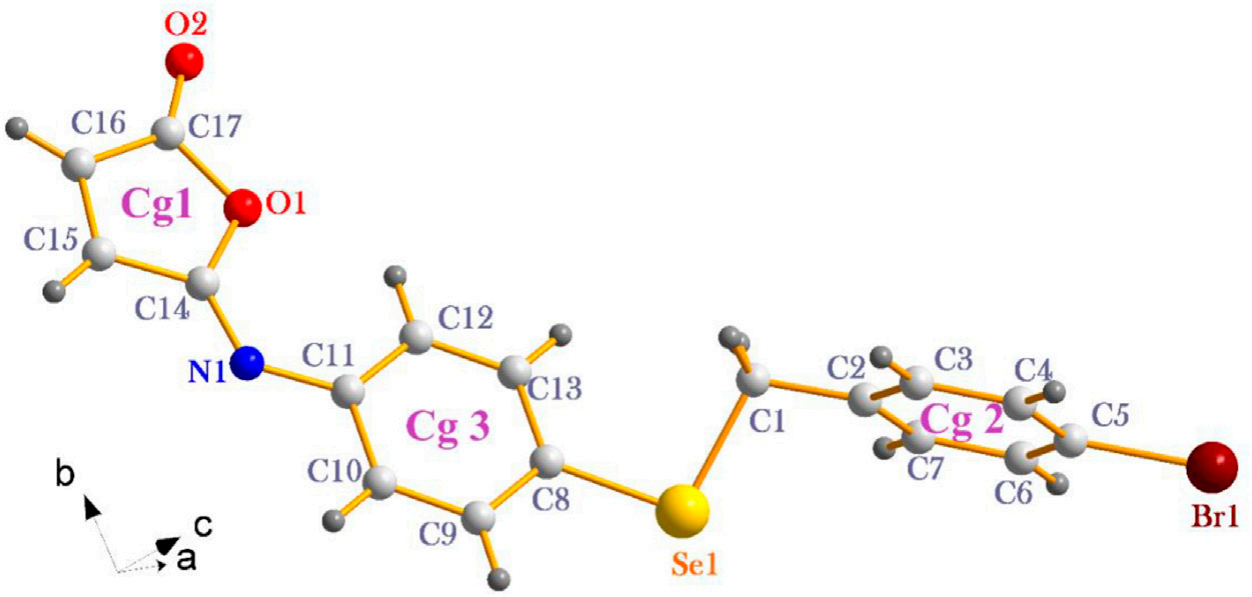
The molecular structure of isomaleimide **4** with atomic labeling.

The isomaleimide **4** crystallized in the triclinic with space group P-1. Its molecular structure is shown in Figure 1. The main body of the (A) structure consists of a Furan ring (Cg1) which has an α,β-unsaturated carbonyl group, and two benzene rings (Cg2 and Cg3). The planarity of the Cg1 (C14-C17-O1/O2), Cg2 (C2-C7/Br1/C1), and Cg3 (C8-C13/Se1) is evident by the root-mean-square (rms) deviation of 0.0096 of Cg1, 0.0065 of Cg2, and 0.0084 Å of Cg3. The dihedral angle between Cg2 and Cg3 is 72.32 (9)°. The central Se atom shows a bent geometry [C1-Se1-C8 = 98.29 (10)°]. All bond distances and angles in isomaleimide **4** are within the acceptable ranges (Table 2) (Allen, 2002; Gouda et al., 2022; Shaaban et al., 2022).

**TABLE 2:** Selected bond distances and angles of isomaleimide **4.**

Bond distances (Å)			
C1-C2	1.500 (3)	C9-C10	1.390 (3)
C1-Se	1.968 (2)	C10-C11	1.401 (3)
C2-C7	1.391 (4)	C11-C12	1.400 (3)
C2-C3	1.391 (4)	C11-N	1.414 (3)
C3-C4	1.386 (3)	C12-C13	1.387 (3)
C4-C5	1.386 (3)	C14-N	1.266 (3)
C5-C6	1.381 (3)	C14-O2	1.389 (3)
C5-Br	1.903 (2)	C14-C15	1.459 (3)
C6-C7	1.388 (3)	C15-C16	1.331 (3)
C8-C13	1.396 (3)	C16-C17	1.469 (3)
C8-C9	1.401 (3)	C17-O1	1.195 (3)
C8-Se	1.913 (2)	C17-O2	1.405 (3)
Bond Angles (°)			
C2-C1-Se	108.13 (16)	C9-C10-C11	120.9 (2)
C7-C2-C3	118.5 (2)	C12-C11-C10	118.8 (2)
C7-C2-C1	121.5 (2)	C12-C11-N	126.6 (2)
C3-C2-C1	120.0 (2)	C10-C11-N	114.6 (2)
C4-C3-C2	121.3 (2)	C12-C13-C8	120.6 (2)
C3-C4-C5	118.7 (2)	N-C14-O2	126.1 (2)
C6-C5-C4	121.4 (2)	N-C14-C15	125.9 (2)
C6-C5-Br	119.52 (18)	O2-C14-C15	107.99 (19)
C4-C5-Br	119.07 (18)	C16-C15-C14	108.7 (2)
C5-C6-C7	119.0 (2)	C15-C16-C17	108.1 (2)
C6-C7-C2	121.0 (2)	O1-C17-O2	120.0 (2)
C13-C8-C9	119.3 (2)	O1-C17-C16	132.6 (2)
C13-C8-Se	122.20 (17)	O2-C17-C16	107.4 (2)
C9-C8-Se	118.47 (16)	C14-N-C11	126.4 (2)
C10-C9-C8	119.8 (2)	C14-O2-C17	107.77 (18)
C13-C12-C11	120.4 (2)	C8-Se-C1	98.29 (10)

**FIGURE 2:**
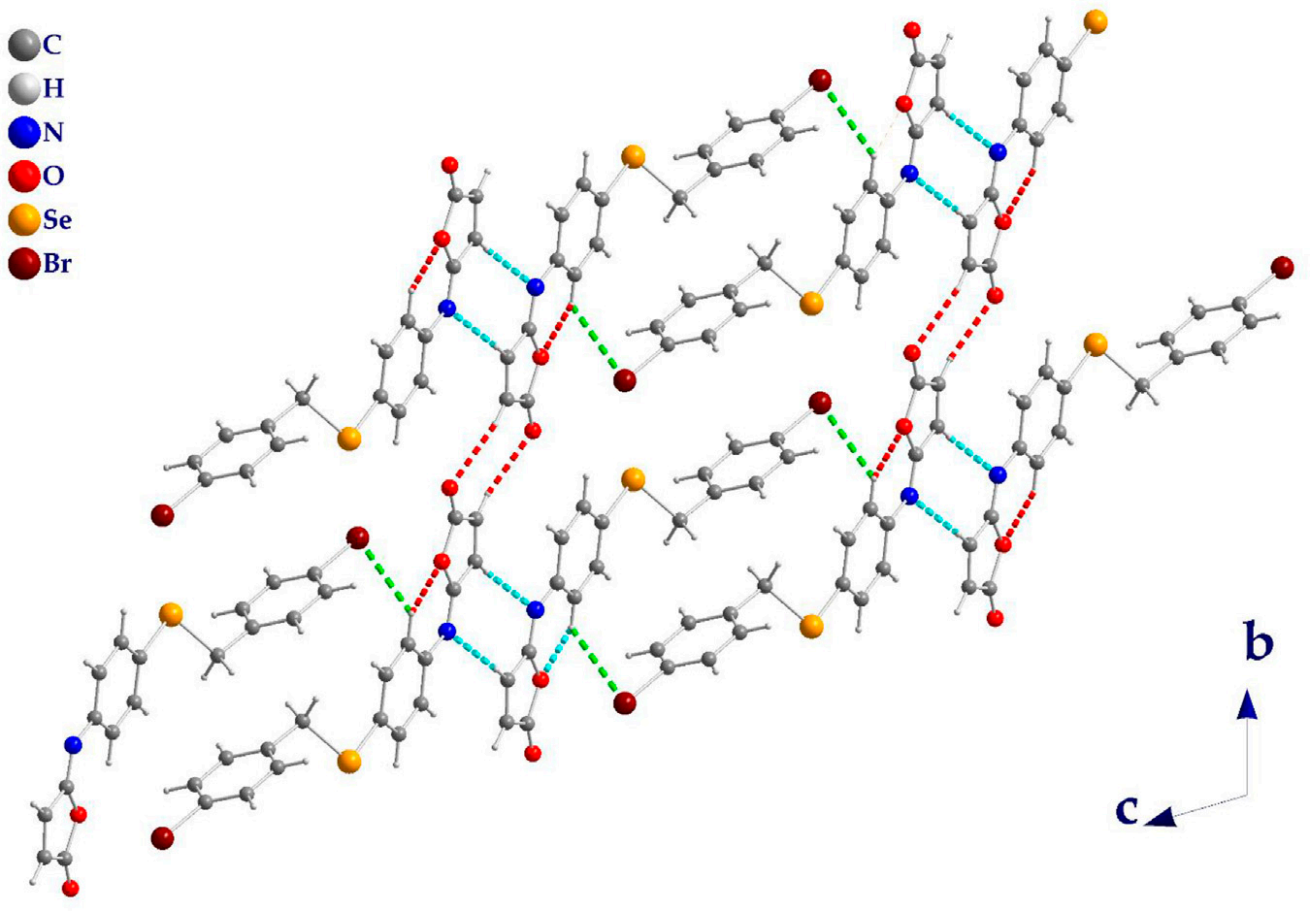
Isomaleimide **4** packing diagram, viewed along *a* axis, showing the hydrogen-bonded chains structure (dashed line). C-H···O (Red), C-H···N (Cyan), and C-H···Br (Green).

**FIGURE 3:**
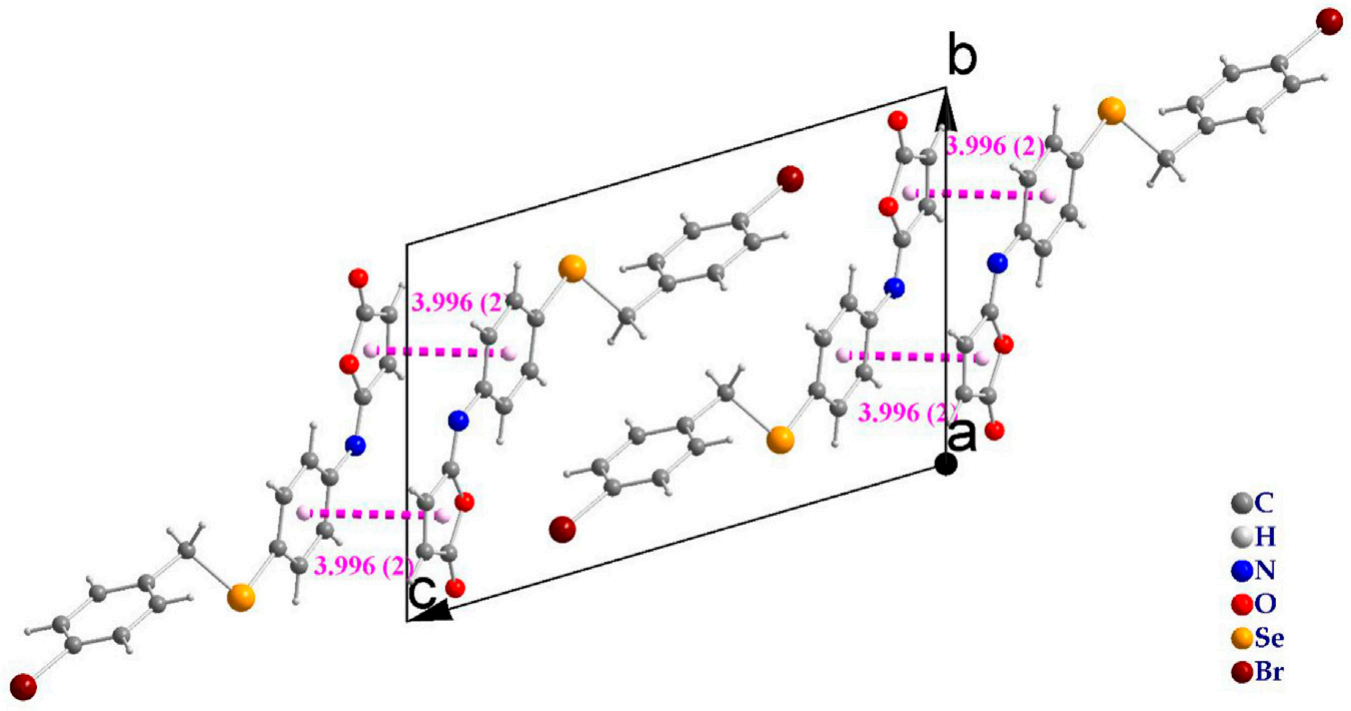
Slipped π-stacked dimers of isomaleimide **4**.

**FIGURE 4:**
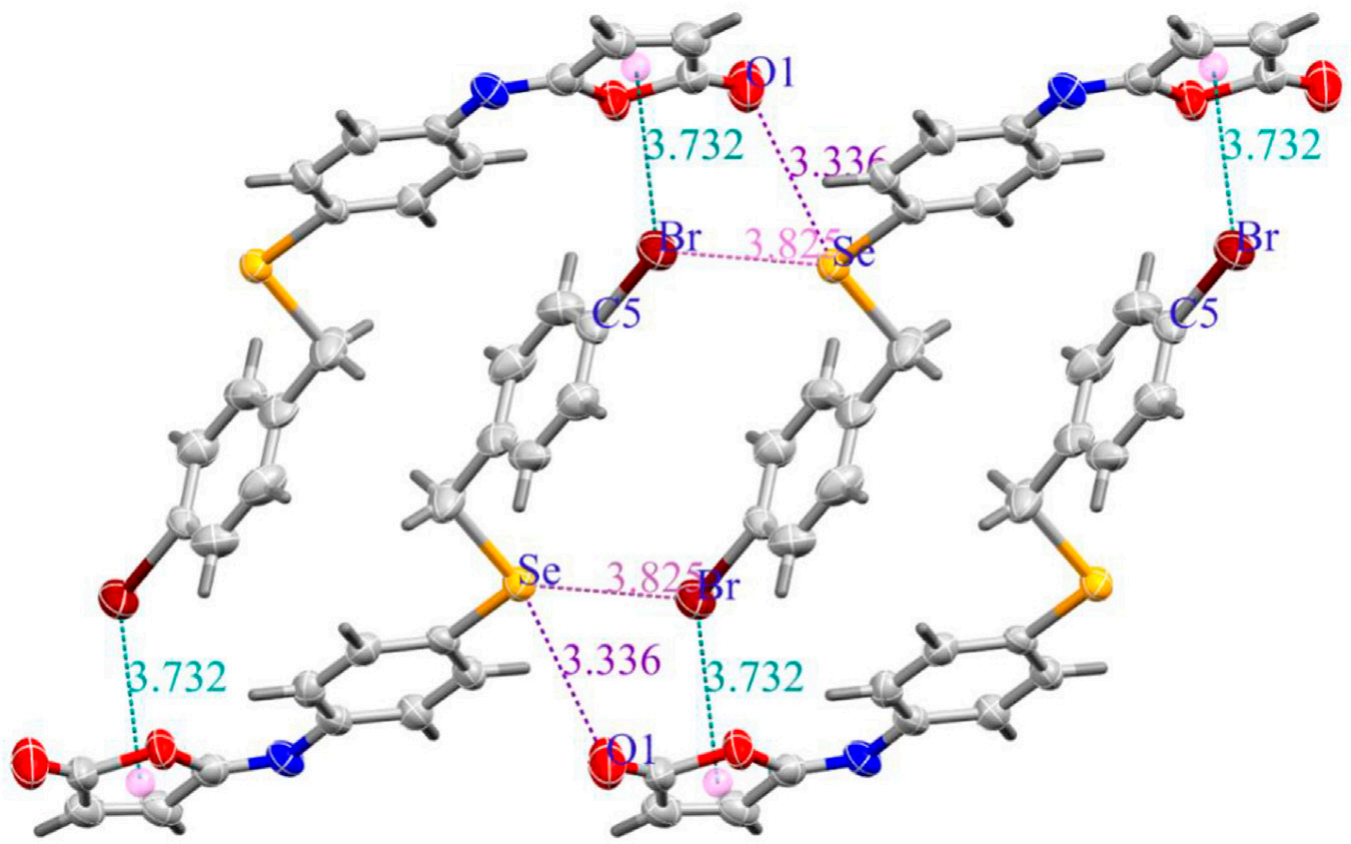
(a) C-Br···Cg (Green dashed lines), Se···O (Purple dashed lines), and Se···Br (Pink dashed lines) interactions in isomaleimide **4**.

The molecules in isomaleimide **4** are linked together by weak non-classical Hydrogen-bonding chains (C16-H16···O1^iii^ and C15-H15···N^ii^ [symmetry codes: (ii) −x−1, −y+1, −z; (iii) −x, −y+2, −z]), (Figure 2; Table 3). It is worth noting that the proximity of donor atoms C12 and acceptor atom O2 allows for the formation of one intramolecular hydrogen bond C12-H12···O2. Bromine atoms play an essential role in crystal packing stabilization because they are involved in C12-H12···Br^i^ hydrogen bonding [symmetry code: (i) −x+2, −y+1, −z+1], (Figure 2). The main characteristics of the hydrogen bonds data are listed in Table 3. Slipped π-stacking interactions strengthen the chains of molecules along *a* axis. The centroid-to-centroid separation of Cg1 and Cg3 is 3.564(1) Å (Figure 3; Table 3). The crystal packing of 4 is also dominated by C-Br···Cg1 (d_Br···Cg1_ = 3.7321(11) Å), Se···Br and Se···O interactions (Figure 4; Table 3). The closest intermolecular Se···Br distance is 3.8251(5) Å, which is greater than the sum of the van der Waals radii for these two elements (3.75 Å) (Bondi, 1964).

**TABLE 3:** Hydrogen bonds, Y-X...Cg interactions, π···π interactions parameters for isomaleimide **4**.

*D*-H···*A*	*D*-H (Å)	H···*A* (Å)	*D*···*A* (Å)	*D*-H···*A* (°)
C12-H12···O2	0.95	2.23	2.857 (3)	123
C12-H12···Br^i^	0.95	3.06	3.834 (2)	140
C15-H15···N^ii^	0.95	2.48	3.389 (3)	161
C16-H16···O1^iii^	0.95	2.40	3.320 (3)	162
	**Y···X (Å)**	**d(X···Cg1) (Å)**	**d(Y···Cg) (Å)**	**Y-X···Cg**
C5-Br···Cg1^iv^	1.903 (2)	3.7321 (11)	4.553 (3)	102.92 (7)
	**Cg(I) ···Cg(J)**	**α**	**Cg(I)perp**	**Cg(J)perp**
Cg1···Cg3^(v)^	3.5641 (14)	3.63 (12)	3.2730(10)	3.3491(9)

Symmetry codes: (i) −x+2, −y+1, −z+1; (ii) −x−1, −y+1, −z; (iii) −x, −y+2, −z; (iv) 1−x,1−y, 1−z; (v) −x, 1−y, −z. Cg(I) = Plane number I, Cg(J) = Plane number J, Alpha = Dihedral Angle between Planes I and J (Deg), CgI_Perp = Perpendicular distance of Cg(I) on ring J (Ang.), CgJ_Perp = Perpendicular distance of Cg(J) on ring I (Ang.).

### 3.5 Hirshfeld surfaces, fingerprint plots, and energy framework

**FIGURE 5:**
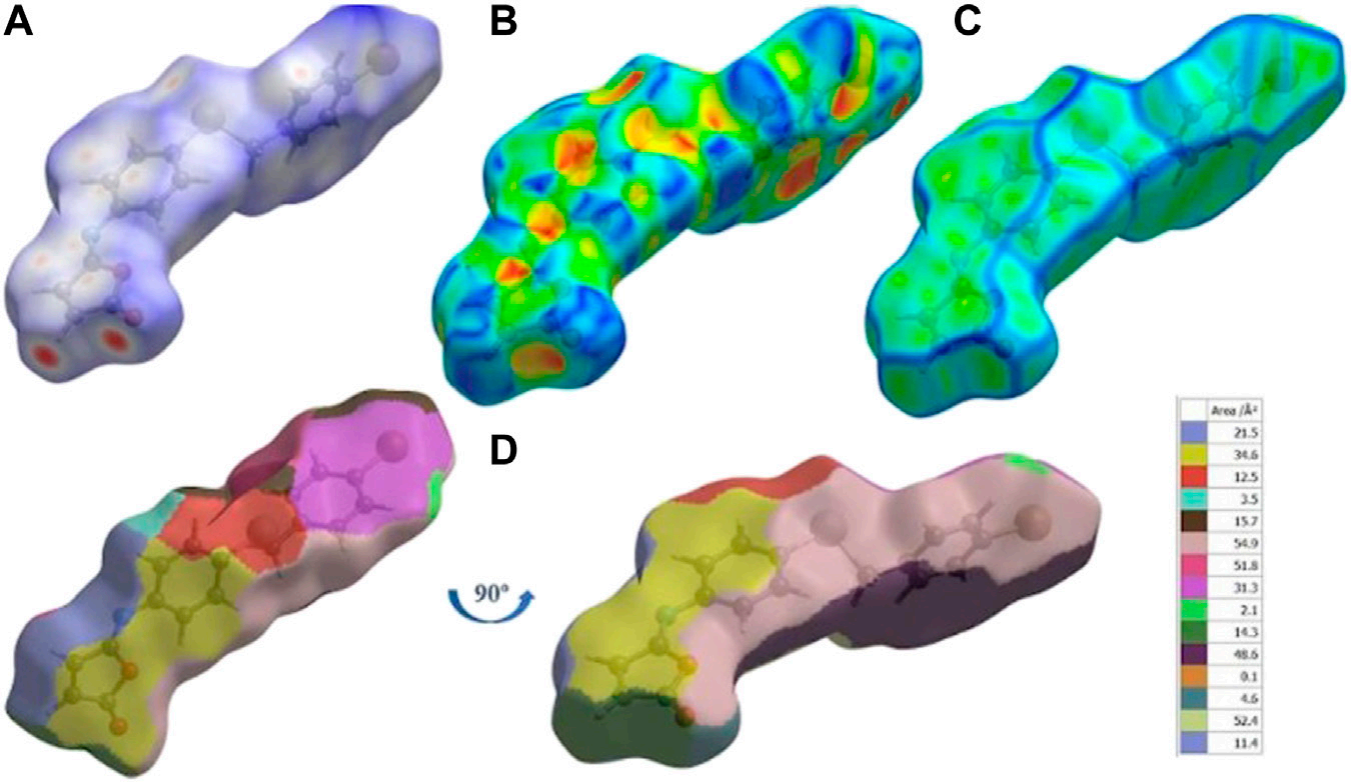
**(A)** d_norm_ mapped on Hirshfeld surfaces for visualizing the intermolecular interactions, **(B)** shape-index, **(C)** curvedness, and **(D)** fragment patches of isomaleimide **4**.

The Hirshfeld surface of isomaleimide **4** is mapped over d_norm_ **(** Figure 5A) in the ranges −0.2503 to 1.4928 Å, shape index (Figure 5B) in the range (−0.9976 to 0.9980 Å), curvedness (Figure 5C) in the range −4.1687 to 0.2406 Å and fragment patches (Figure 5D) in the range 0.000–14.000 Å.

Around the C.H. and the carbonyl group in the Furan ring and the N-atom of isomaleimide **4**, the H.S. has intense red spots, which indicates that these specific atoms are promising in the H-bonding interactions (C15-H15···N and C16-H16···O1). By plotting H.S., we can not only visualize H-bonding interactions but also investigate other non-covalent interaction types, such as stacking interactions. To visualize this interaction, the H.S. is plotted on a shape index and curvedness. The presence of triangular regions of red and blue around the furan and aromatic rings on the shape index (Figure 5B) and a flat region around the phenyl rings and furan rings in curvedness (Figure 5C) indicates the presence of π-stacking interactions. The nearest molecules’ neighbor environment is determined by the color patches (Figure 5D) on the Hirshfeld surface and their proximity to adjacent molecules.

**FIGURE 6:**
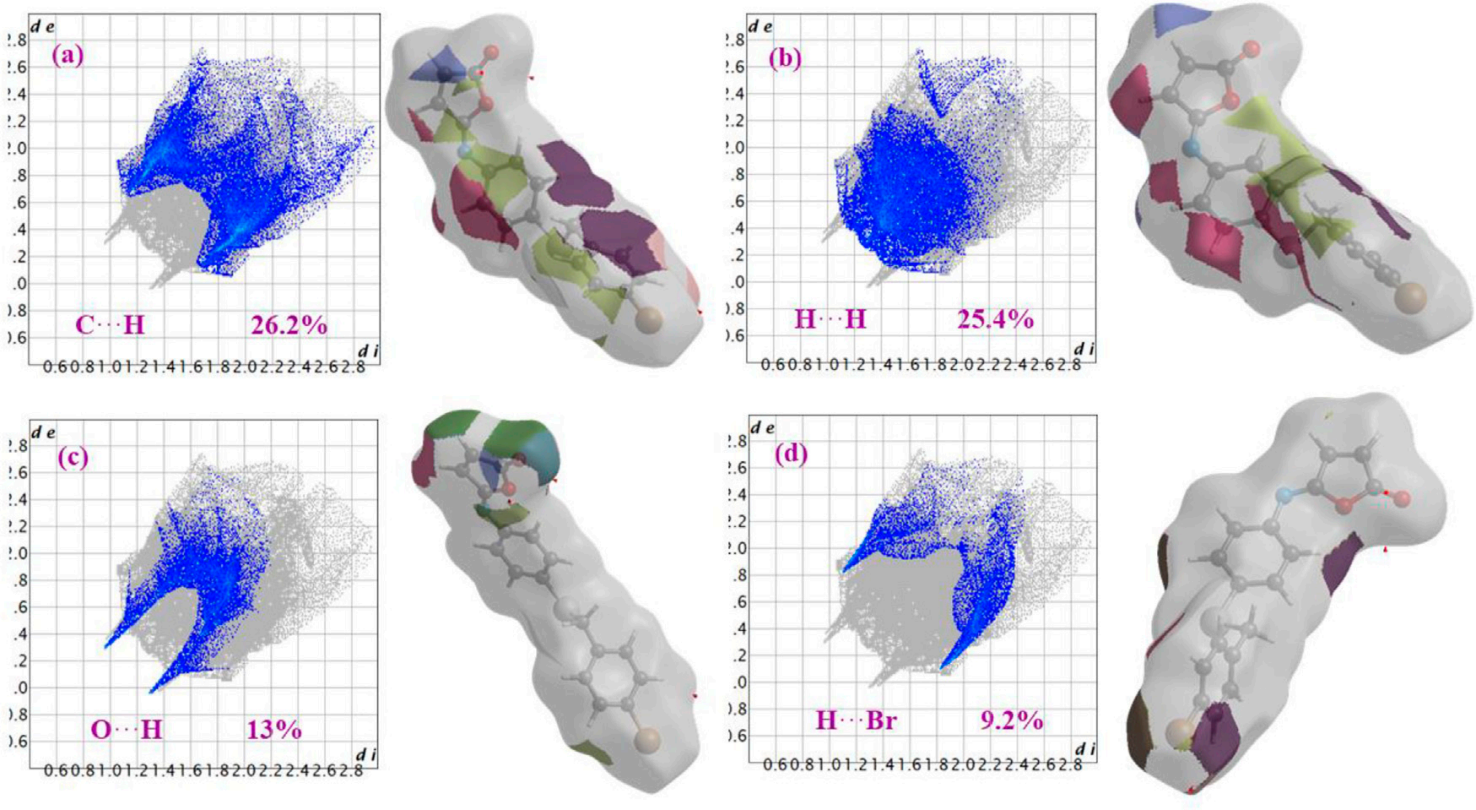
Fingerprint plots with fragment patches of **(a)** C∙∙∙H, **(b)** H∙∙∙H, **(c)** O∙∙∙H and **(d)** H∙∙∙Br contacts in isomaleimide **4**.

Figure 6 shows the two-dimensional fingerprint of the significant contacts contributing to the Hirshfeld surface of isomaleimide **4** is showed in expanded mode. Interatomic contact percentage contributions in isomaleimide **4** are calculated using two-dimensional fingerprint plots. The C∙∙∙H/H∙∙∙C contacts contribute the most (26.2%), corresponding to C-H···N and C-H···Br interactions, as shown in the 2D fingerprint plot by a pair of pointed spikes representative of strong hydrogen-bonding interactions (Figure 6A).

**FIGURE 7:**
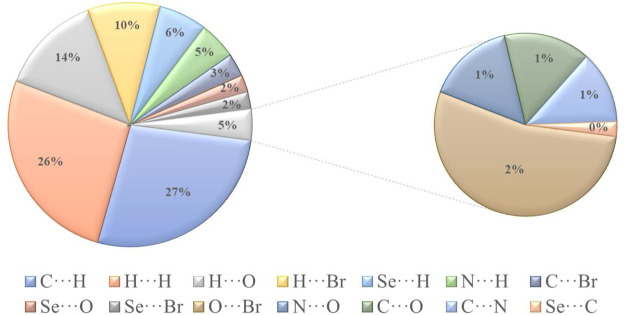
Different intermolecular contacts’ relative contributions to the Hirshfeld surface area in isomaleimide **4.**

The H∙∙∙H contact appears in the middle of the scattered points in the two-dimensional fingerprint plots (Figure 6B) with a contribution to the overall Hirshfeld surface of 25.4%. Because of the abundance of hydrogen on the molecular surface, they are the second most common interactions (55%). O∙∙∙H/H∙∙∙O contacts make up the third-largest contribution to the Hirshfeld surface (Figure 6C), accounting for 13%. This contact indicates the presence of intermolecular C-H···O hydrogen bonds. The Br∙∙∙H/H∙∙∙Br contacts (Figure 6D), which raise to N-H∙∙∙Br interactions, are the third most crucial interaction on the surface, accounting for approximately 9.2% of the Hirshfeld surfaces. The relative percentage contributions to the overall Hirshfeld surface are shown in Figure 7. Finally, the Hirshfeld surface analysis yields the same results as the X-ray crystal structure analysis and provides a new visual explanation for intermolecular interactions.

**FIGURE 8:**
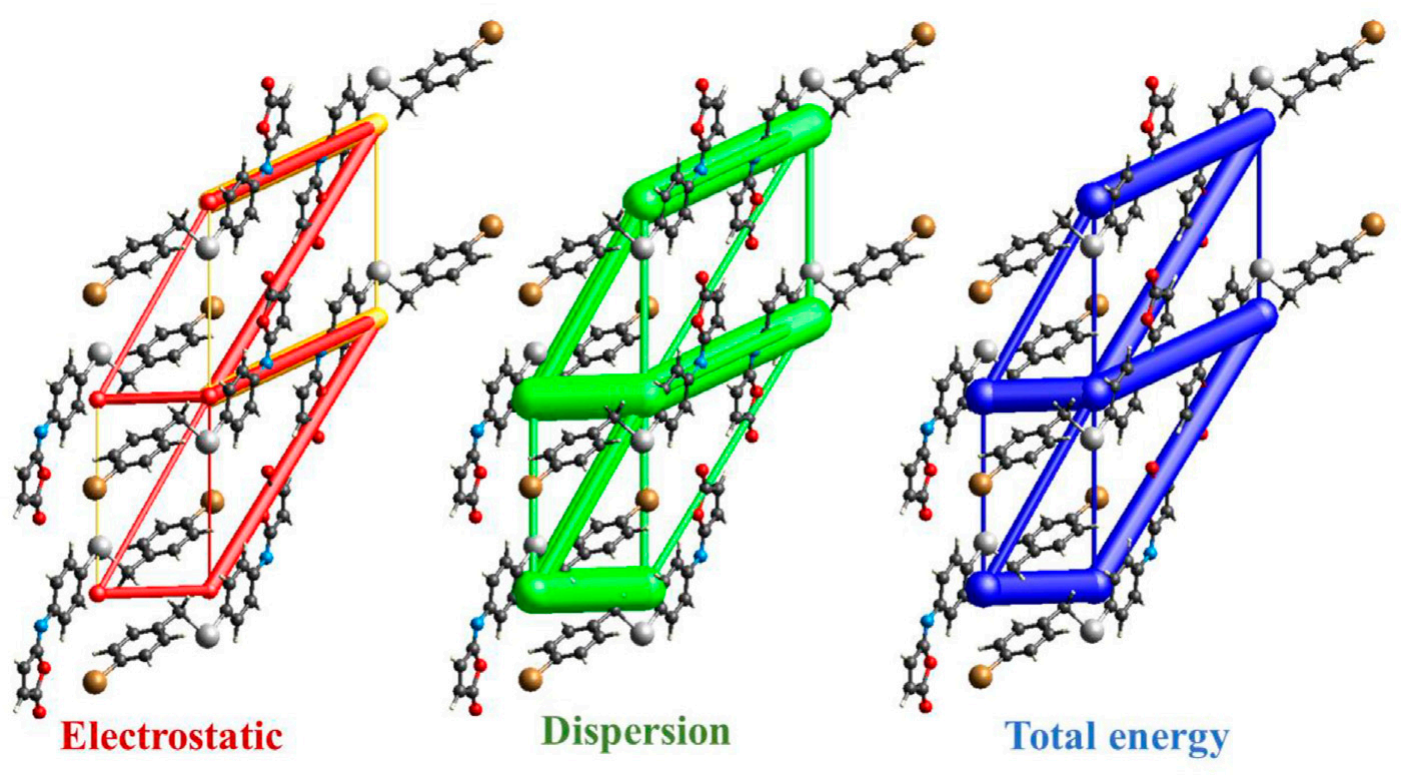
Energy-framework diagrams of isomaleimide **4** along the *a* axis: Electrostatic interaction energies (red); dispersion interaction energies (green); total interaction energies (blue).

The intermolecular interaction energies are calculated using the HF/3-21G energy model with scale factors to determine E_tot_: k_ele_ = 1.019, k_pol_ = 0.651, k_dis_ = 0.901, k_rep_ = 0.811, where a cluster of molecules is generated by applying crystallographic symmetry operations with respect to a chosen central molecule within a radius of 3.8 by default. The total interaction energies (E_tot_ = −263 kJ/mol) can be divided into total electrostatic (E_ele_ = −54.7 kJ/mol), total polarization (E_pol_ = −33.6 kJ/mol), total dispersion (E_dis_ = −327 kJ/mol), and total repulsion (E_rep_ = 137 kJ/mol) (Table 4). Obviously, the interactions between neighboring molecules contribute the most to the stability of this structure, and in most cases, the dispersion energy component dominates these interactions. Additionally, even for the hydrogen-bonded molecule pairs, the dispersion energy component is dominant or comparable to the electrostatic components which due to the presence of π-π and C-Br···π interactions (the highest importance of electrostatic components is observed in a pair having E_ele_ = −22.6 kJ mol^−1^, E_pol_ = −4.5 kJ mol^−1^ and E_disp_ = −54.8 kJ mol^−1^). Figure 8 depicts the Coulomb interaction energy (red), dispersion energy (green), and total interaction energy (blue) between molecular pairs along the *a* axis with respect to the selected molecule.

**TABLE 4:** Interaction energies (kJ mol^−1^) calculated for isomaleimide **4**.

N	Symop	R	Electron density	E_elec	E_pol	E_dis	E_rep	E_tot
2	x, y, z	9.89	HF/3-21G	4.5	−1.6	−11.8	6.4	−1.9
2	x, y, z	10.18	HF/3-21G	−3.1	−0.8	−2.6	0.1	−0.6
1	−x, −y, −z	8.85	HF/3-21G	−0.4	−1.0	−17.3	7.6	−10.5
1	−x, −y, −z	12.43	HF/3-21G	27.9	−4.2	−60.9	0.0	−29.2
2	−x, −y, −z	15.71	HF/3-21G	−18.1	−7.0	−20.1	0.0	−41.0
2	x, y, z	5.79	HF/3-21G	−22.6	−4.5	−54.8	34.2	−47.6
1	−x, −y, −z	4.73	HF/3-21G	−8.4	−3.4	−61.5	32.0	−40.2
1	−x, −y, −z	19.64	HF/3-21G	−20.1	−5.8	−10.6	0.0	−33.8
1	−x, −y, −z	10.76	HF/3-21G	−8.0	−1.2	−32.3	26.7	−16.4
1	−x, −y, −z	5.68	HF/3-21G	−13.4	−4.3	−55.1	30.0	−41.8

E: interaction energies components, Symop: rotational symmetry operations with respect to the reference molecule, R: the centroid-to-centroid distance between the reference molecule N: interacting molecules, and the number of pair(s) of interacting molecules with respect to the reference molecule (Sheldrick, 2015).

### 3.6 Density functional theory calculations

#### 3.6.1 Geometric structures

**FIGURE 9:**
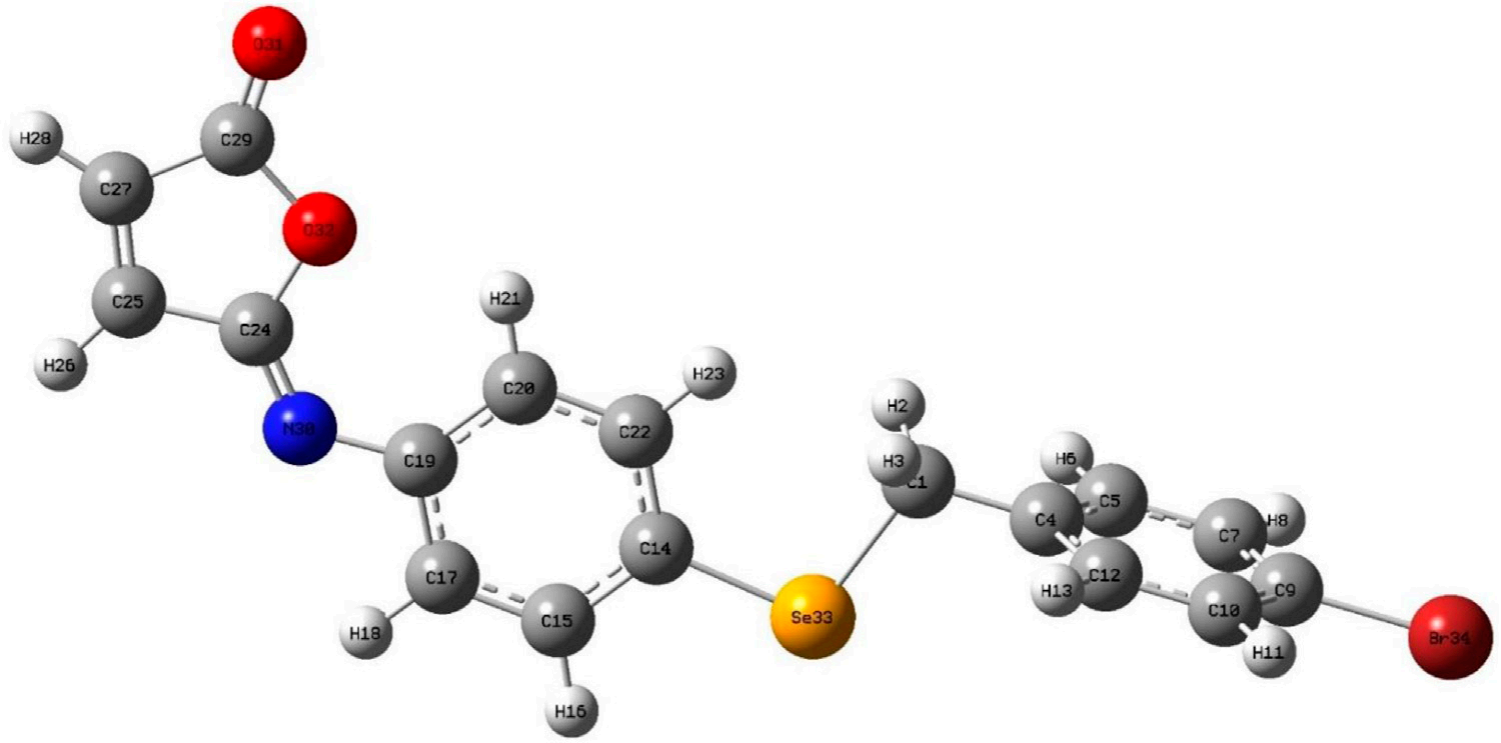
Optimized molecular structure of isomaleimide **4.**

**FIGURE 10:**
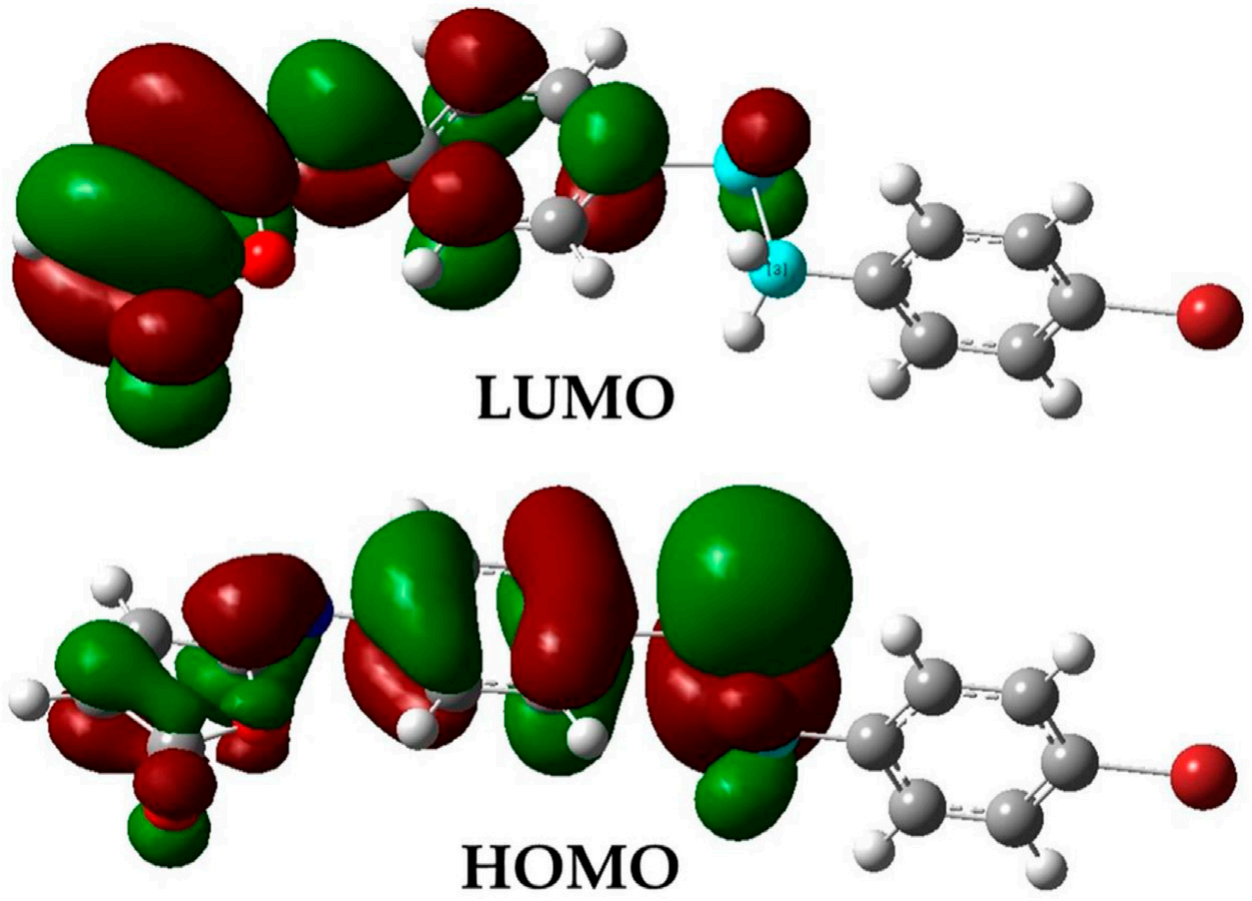
Electronic distribution of LUMO and HOMO molecular orbitals of the isomaleimide **4**.

The optimized structural parameters of the isomaleimide **4** are depicted in Figure 9. In this theoretical study, the hybrid functional B3LYP with the basis 6-311 ++ G(d,p) has been used in all the calculations made by the Gaussian 09 program. The optimization of the structure of the studied compound has been carried out, starting from the geometry of X-rays. A comparison of experimental results with theoretical ones (Table 5) reveals that most of the calculated values of bond lengths and angles are very close to those experimental data, which shows that the choice of the base 6-311 ++ G(d,p) is suitable for this theoretical study. However, the slight difference observed can be attributed to the environment of the molecule studied, being isolated in phase gaseous for the theoretical study and subjected to interactions with solid-state intermolecular molecules in the experimental study. Figure 10 depicts the visual HOMO and LUMO of compound **4**.

**TABLE 5:** Geometric parameters obtained using DFT/B3LYP//6-311 ++ G(d,p).

Distance	X-ray	DFT	Valence angles	X-ray	DFT
C1-Se	1.968 (2)	1.998	C4-C1-Se	108.13 (16)	109.10
C9-Br	1.903 (2)	1.916	C17-C19-N	114.6 (2)	115.19
C14-Se	1.913 (2)	1.923	C20-C19-N	126.6 (2)	126.71
C19-N	1.414 (3)	1.394	C7-C9- Br	119.07 (18)	119.46
C24-N	1.266 (3)	1.269	C10-C9-Br	119.52 (18)	119.47
C24-O32	1.389 (3)	1.398	C15-C14-Se	118.47 (16)	116.85
C29-O32	1.405 (3)	1.406	C22-C24-Se	122.20 (17)	124.16
C29-O31	1.195 (3)	1.194	N-C24-C25	125.9 (2)	125.36
C4-C5	1.391 (4)	1.399	N-C24-O32	126.1 (2)	126.84
C4-C12	1.391 (4)	1.399	O32-C24 C25	107.99 (19)	107.78
C15-C17	1.390 (3)	1.383	O32-C29-C27	107.4 (2)	106.87
C10-C12	1.386 (3)	1.392	O31-C29-C27	132.6 (2)	131.58
C5-C10	1.386 (3)	1.391	O31-C29-O32	120.0 (2)	121.54
C17-C19	1.401 (3)	1.408	C24-N-C19	126.4 (2)	127.72
C20-C22	1.387 (3)	1.390	C24-O32-C29	107.77 (18)	108.48
C24-C25	1.459 (3)	1.459	C14-Se-C1	98.29 (10)	101.25

Furthermore, we believe it is worthwhile, to begin with the results gained from the geometrical parameters of the investigated molecules. In Table 4, the results of this calculation are grouped by the following numbering scheme. Compare the experimental results from a crystallographic investigation with these theoretically computed geometric characteristics (Klocker et al., 2003). The relation:

$$\Delta =\frac{\left|X_{\mathrm{theo}}-X_{\exp}\right|}{X}\times 100$$ (1)

Where, the theoretical value of the quantity *X* is *X*_*theo*_, while the experimental value is *X*_*exp*_. The average variance of the distances and angles produced by the DFT approach is on the order of less than 2%, indicating that the diverse theoretical conclusions obtained are in excellent agreement with those found experimentally by crystallographic analysis.

#### 3.6.2 Study of overall global reactivities

The amount of energy required to remove an electron from a molecule is known as ionization potential. Furthermore, high ionization energy denotes great stability and thus chemical inertness, whereas low ionization energy shows a molecule’s reactivity. The energy generated when an electron is introduced to a neutral molecule is characterized as electron affinity isomaleimide **4**. A significant value isomaleimide **4** shows the molecule’s tendency to keep its electrons. A negative chemical potential (*μ*) reflects the molecule’s molecular stability or difficulty breaking it down into its constituent parts (Table 6). The resistance of the cloud of molecular electrons to deformation during tiny perturbations is measured in hardness (*η*). A big HOMO-LUMO energy gap implies a complicated molecule with low polarizability and chemical and biological activities but high kinetic sensitivity (Table 6).

**TABLE 6:** Global indices of the reactivity isomaleimide **4**.

Electronic energy (eV)	−158809.58
E_HOMO_ (eV)	−5.97
E_LUMO_ (eV)	−3.07
Gap, ∆E	2.90
Dipole moment, (Debye)	2.18
Chemical potential (eV)	−4.52
Electronegativity	4.52
Hardness	1.45
Softness	0.73
Global softness	0.69
Electrophilicity index	7.04

In contrast, a small HOMO-LUMO energy gap indicates a soft molecule with high polarizability and activities but low kinetic sensitivity. Chemical and biological sensitivities are low, whereas kinetic sensitivity is heightened. The overall electrophilicity index (*ω*) of a molecule measures its stabilization energy or resistance to exchange electrons with the system after the addition of an external electronic charge (Parr et al., 1999).

The gap energy, which is the energy difference of the two preceding molecular orbitals (E_g_ = E.L.U.M.O.–E.H.O.M.O.) is 2.90 eV. Chemical potential, electronegativity, hardness, softness, Global Softness, and electrophilicity for the isomaleimide **4** were determined as −4.52, 4.52, 1.45, 0.73, 0.69, and 7.04 eV, respectively, using Eqs 2–7 (Rajan and Muraleedharan, 2017):

$$x=-1/2\left(E_{\mathrm{LUMO}}+E_{\mathrm{HOMO}}\right)$$ (2)

$$\mathrm{\mu =-x}=1/2\left(E_{\mathrm{LUMO}}+E_{\mathrm{HOMO}}\right)$$ (3)

$$\eta =1/2\left(E_{\mathrm{LUMO}}-E_{\mathrm{HOMO}}\right)$$ (4)

S=1/2η (5)

$$\omega =\mu^{2}/2\eta$$ (6)

σ=1/η (7)

#### 3.6.3 Surfaces with molecular electrostatic potential

**FIGURE 11:**
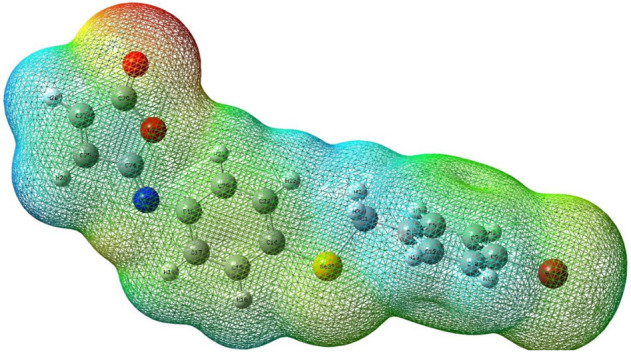
Molecular electrostatic potential (M.E.P.) map of isomaleimide **4** calculated at the 6-311++G(d,p) level.

Isomaleimide **4**’s M.E.P. was computed using the DFT-B3LYP/6-31G ++ optimized geometry, and its surface map is shown in Figure 11. This diagram uses a color scheme to indicate the electrostatic potential values. The highest negative value is represented by the red color, indicating the most likely areas for electrophilic assault. The most positively charged areas appear in dark blue color, indicating potential nucleophilic attack sites. The determined limits are −4.64e-2 (deepest red) and +4.64e-2 (deepest blue), with the intermediate color scale flowing from red through orange, yellow, green, and blue in that order, as illustrated in Figure 11. The most substantial negative potential is centered near the oxygen atoms, while positive potentials are scattered throughout the TTF unit, particularly around the outer H atoms. Finally, the M.E.P. primarily suggests an electrophilic attack on oxygen atoms, with the possibility of a nitrogen atom attack. On the other hand, a strong base may be able to destroy one of the H atoms as a proton.

## 4 Conclusion

Isomaleimide **4** was accidentally obtained in 77% yield instead of the maleimide **5** *via* dehydration and cyclization of the respective *N*-maleanilic acid **3** upon heating with acetic anhydride. The molecular structure of isomaleimide **4** was confirmed by X-ray diffraction analysis. The cytotoxicity was assessed against two oligodendrocytes and the antioxidant properties were evaluated using H2-DCFDA assay. The intermolecular interactions in the crystal packing are quantified and visualized using Hirshfeld surfaces, 2D fingerprint plots, and 3D energy frameworks. The two-dimensional fingerprint revealed that the most significant contributions to these surfaces come from C∙∙∙H/H∙∙∙C (26.2%), H···H (25.4%), O∙∙∙H/H∙∙∙O (13%) and Br∙∙∙H/H∙∙∙Br (9.2%) interactions. The energy-framework analysis reveals that the dispersive energies are the most important forces in the crystal. In parallel with the experimental study, we carried out a theoretical study detail using quantum chemical methods to determine the properties structural of compound **4**. We carried out a comparison between the theoretical geometrical parameters and those obtained by X-ray diffraction. It appears significantly that the calculations obtained are in good agreement with the experimental data.

## Data Availability

The original contributions presented in the study are included in the article/Supplementary Material, further inquiries can be directed to the corresponding authors.
