# Supplementary material for: Unexpected kinetically controlled organoselenium-based isomaleimide: X-ray structure, hirshfeld surface analysis, 3D energy framework approach, and density functional theory calculation
Source: Front Chem. 2022 Aug 5;10:961787. doi: 10.3389/fchem.2022.961787 (PMC9388736; doi:10.3389/fchem.2022.961787)
Supplement: Supplementary file 1 [file DataSheet1.docx]

Unexpected Kinetically Controlled Organoselenium-Based Isomaleimide: X-Ray Structure, Hirshfeld Surface Analysis, 3D Energy Framework Approach, and DFT Calculation

Saad Shaaban^1,2^*, Hela Ferjani ^3^, Hany M. Abd El-Lateef ^1,4^, Mai M. Khalaf ^1,4^, Mohamed Gouda ^1^, Mohamed Alaasar ^5,6,^*, and Tarek A. Yousef ^3,7,^*

^1^ Department of Chemistry, College of Science, King Faisal University, P.O. Box 380, Al-Ahsa 31982, Saudi Arabia; sibrahim@kfu.edu.sa (S.S.); hmahmed@kfu.edu.sa (H.M.A.E.0L.); mmkali@kfu.edu.sa (M.M.K.); mgoudaam@kfu.edu.sa (M.G.)

^2^ Department of Chemistry, Organic Chemistry Division, College of Science, P.O. Box 11432,
Mansoura University, Mansoura 11001, Egypt; dr_saad_chem@mans.edu.sa (S.S.)

^3^ Department of Chemistry, College of Science, IMSIU (Imam Mohammad Ibn Saud Islamic University), P.O. Box 5701, Riyadh 11623, Saudi Arabia; hhferjani@imamu.edu.sa; tayousef@imamu.edu.sa (T.A.Y.)

^4^ Chemistry Department, Faculty of Science, Sohag University, P.O. Box 82524, Sohag, Egypt;
hmahmed@kfu.edu.sa

^5^ Institute of Chemistry, Martin Luther University Halle-Wittenberg, Kurt Mothes Str. 2, D-06120 Halle (Saale), Germany, mohamed.alaasar@chemie.uni-halle.de

^6^ Department of Chemistry, Faculty of Science, Cairo University, Giza, Egypt

^7^ Toxic and Narcotic Drug, Forensic Medicine Department, Mansoura Laboratory, Medicolegal Organization, Ministry of Justice, P.O. Box 12432, Cairo 11435, Egypt; tayousef@imamu.edu.sa (T.A.Y.)

***** Correspondence: sibrahim@kfu.edu.sa or dr_saad_chem@mans.edu.sa (S.S.); mohamed.alaasar@chemie.uni-halle.de (M.A.); tayousef@imamu.edu.sa (T.A.Y.)

1. **Material and methods**

All chemicals were obtained from Sigma. Solvents were dried prior to use. The spectroscopic studies were measured at the “Pôle Chimie Moléculaire” de l'Université de Bourgogne (PACSMUB)”. The mass of the organic selenides was obtained on an Electro-Spray Ionization Mass Spectrometry (Thermo LTQ Orbitrap XL ESI–MS) and high-resolution mass spectra. ^1^H (300.13 MHz) and ^13^C (75.5 MHz) NMR spectra were analyzed on Bruker 300 Avance III, spectrometers. The values of the chemical shifts (δ) are presented in parts per million relatives to tetramethyl silane, using deuterated solvent as an internal standard. All chemicals were purchased from Sigma e.g., 7-ketocholesterol, and ascorbic acid and vitamin E. More details on the spectroscopic data can be found in the Supporting Information’s. Compound **1**-**4** was synthesized according to a literature reported method [1].

1. **Biological assays**
   1. **Cytotoxicity assay**

Murine oligodendrocytes (158N and 158JP) were seeded at 240,000 cells per 24-well plates and were grown using the Dulbecco’s Modified Eagle Medium supplemented with penicillin antibiotics (1% v/v, streptomycin) and fetal bovine serum (5% v/v) and kept at 37 °C in atmosphere containing 5% CO_2_. The concentration of 7kc and vitamin E, and the time of treatment were chosen based on data obtained on 7kc treated 158N cells. Organoselenium compounds were prepared in DMSO and then diluted with DMEM up to 1 mM.

- 1. **MTT assay[2-5]**

The MTT assay was carried out on 158N and 158JP cells after one day of incubation with 7kc (50µM)/different concentrations (0, 1, 10, 20, 50 and 100 µM) of the organic selenides. In live cells, the MTT is reduced to formazan by the succinate dehydrogenase mitochondrial enzyme. The microplate reader Tecan Sunrise (Tecan, Lyon, France) was used for the reading of the plates at 570 nm. The IC_50_ values and the maximum concentrations without toxicity were estimated for each of the compounds from the respective dose-response curves.

| Table S1. Evaluation of the cytotoxicity and ROS production by MTT and H2-DCFDA assays. | | | | | |
| --- | --- | --- | --- | --- | --- |
| Compounds | Cytotoxicity assay  IC_50_ (µM) | | **H_2_-DCFDA assay**^b^  **(% of control)** | | |
|  | 158N | 158JP | **C1 (10 µM)** | **C2 (20 µM)** | **C3 (50 µM)** |
| 2 | *a* | *a* | 36 ± 8* | 41 ± 2* | 48 ± 2* |
| 3 | 29 | 50 | 42 ± 10* | 36 ± 20* | 31 ± 20* |
| 4 | *a* | *a* | 7 ± 4* | 8 ± 4* | 11 ± 4* |
| 7kc | 37 | 74 | - | - | 48 ± 2* |
| Vitamin E | - | - | 94 ± 5 | | |
| The cytotoxicity was measured after two days of treatment with different concentrations (0, 1, 10, 20, 50 and 100 µM) of the organic selenides using the MTT assay. The IC_50_ was estimated as the mean of two parallel experiments; 7kc was used as a positive control; ^a^Means no growth inhibition was observed at the tested concentration range. ^b^158N Cells were cultured in the presence of different compounds (C1=10; C2= 20; C3=50 µM); Flow cytometry technique was used to evaluate ROS levels via staining cells with H2-DCF; data are shown as mean ± SD and expressed as % control; significance of the difference between the DMSO and compounds. Treated cells are indicated by (Mann–Whitney test; *: P < 0.05); vitamin E (50 µM) was used as a positive control. | | | | | |

- 1. **H2-DCFDA assay[1,2,5]**

ROS overproduction and O_2_^•−^ and were detected with H2-DCFDA assay. Cultured cells were treated with trypsin re-suspended in PBS (10^6^ cells/mL) and kept in the dark for half hour at 37^°^C with H2-DCFDA at 10 μM and cells were analyzed by flow cytometry. The green fluorescence of 2’,7’- dichlorofluorescein resulting from the oxidation of H2-DCFDA was analyzed by flow cytometry and collected through a 520/10-nm band pass filter. Flomax (Partec) or FlowJo (Tree Star Inc.) software were used for the data analysis.

- 1. **Apoptosis status and detection of Sub-G1 cells[1,2,5]**

The analysis of the cell cycle can reveal a Sub-G1 peak, which is representative of apoptotic cells. Cell cycle analysis was detected on 158N cells stained with Propidium Iodide (PI). Flow cytometric analyses were measured on a Galaxy flow cytometer. Cells were prepared by re-suspension in cold ethanol (80%, −20 °C) for 2 hrs.Cells were then washed withphosphate buffer, and re-suspended in phosphate buffer (300 μL) containing PI (80 μg/mL) and RNase (200 μg/mL) and kept for 1 h at 37°C.PBS 2 mL was then added, and Fluorescence of propidium iodide was recorded using a 590 ± 10 nm band filter. The percentage of cells in different cell cycle phase was manually estimated.

**Figure S1**. Anti-apoptotic effect of the organoselenium derivative **3** on oligodendrocytes. The anti-apoptotic effect was measured by cytometry using propidium iodide. The organoselenium compound **3** was used at different concentrations (50, 10, 5, and 1 μM) to evaluate the percentage of cells in Sub-G1 phase during the cell cycle of 158N cells treated with/without 7kc (50 μM) for 24 hours. Data shown are mean ± SD.

1. **Synthesis of the organoselenium compounds 1-4.**

**Scheme 1**. Reagents and conditions: (i) 4,4'-diselanediyldianiline (**1**) (2.5 mmol), bromo-4-(bromomethyl)benzene (3 mmol), EtOH (30 ml), NaBH_4_ (12.5 mmol); (ii) 4-((4-bromobenzyl)selanyl)aniline (**2**) (2.5 mmol), toluene (15 mL), toxilic anhydride (2.5 mmol), 4 h, r.t.; (iii) *N*-maleanilic acid **3** (2.5 mmol), Ac_2_O (8 mL), 250 mg NaOAc, 3 h, 40-50 ^0^C.

- 1. **4-((4-Bromobenzyl)selanyl)aniline (2)**

Compound **1** (344 mg, 1 mmol) and -bromobenzyl bromide (550 mg, 2.2 mmol), Aliquat 336 (45 mg, 5% mol) and sodium tetrahydridoborate (189.15 mg, 5 mmol). Then the reaction was reflux for additional 3 hrs. The progress of the product formation was followed by TLC petroleum ether: EtOAc= 6:1, R_f_ = 0.36, puriﬁed by column silica gel chromatography with petroleum ether: EtOAc = 6:1.5. White solid; Yield: 310.31 mg (91%); mp 156-158 ^o^C. ^1^H NMR (300 MHz, DMSO) δ 7.37 – 7.33 (m, 2H, Ar-H), 7.24 – 7.20 (m, 2H, Ar-H), 7.01 – 6.94 (m, 2H, Ar-H), 6.59 – 6.55 (m, 2H, Ar-H), 3.90 (s, 2H, SeCH_2_); ^13^C NMR (75 MHz, DMSO) δ 146.65, 138.63, 137.04, 131.31, 130.45, 120.32, 116.49, 115.61, 32.50; MS (ESI): *m/z* = found 364.84 [M^+^+Na]; calcd. 340.93 [M^+^]; HRMS calcd. for C_13_H_12_BrNSe [M^+^+1]: 341.93911, found 341.93760 [M^+^+1].

**
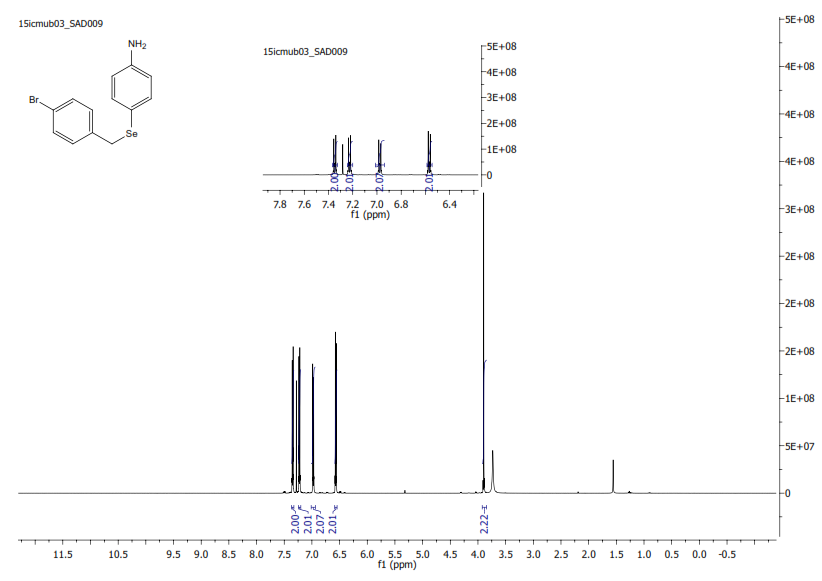
**

**
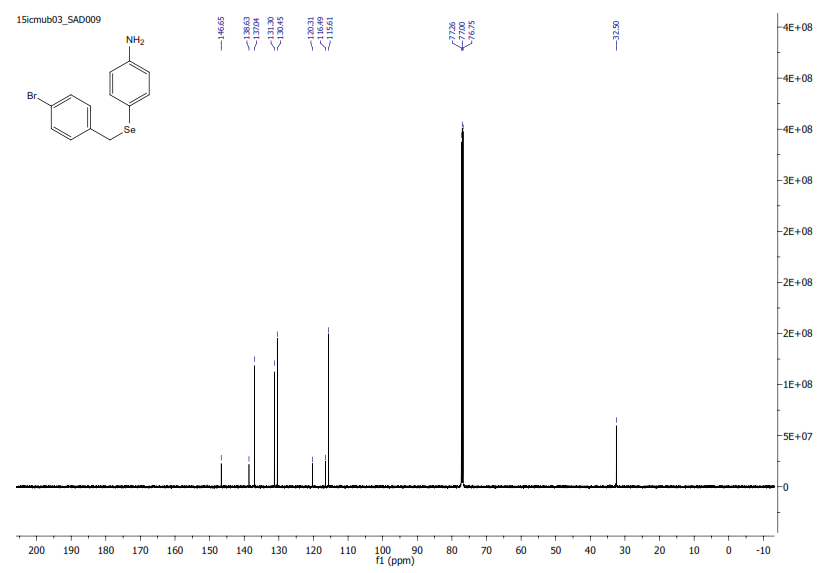
**

**
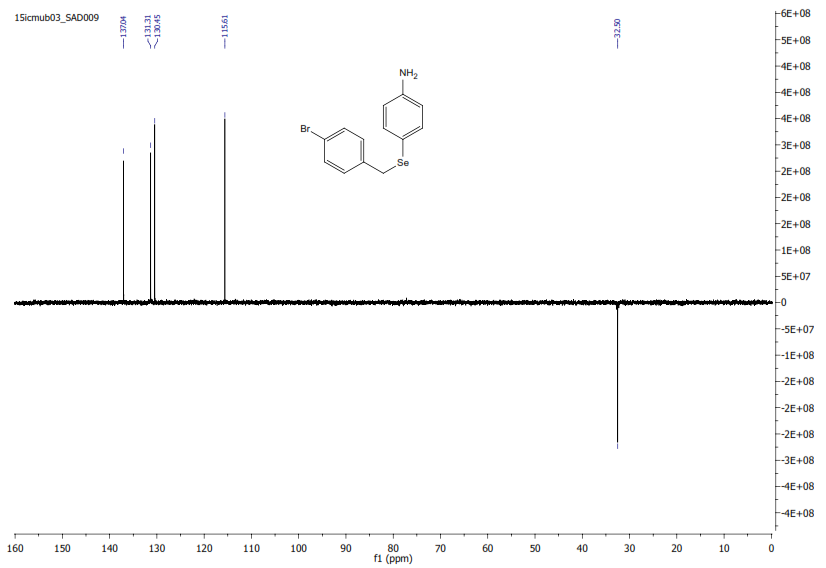
**


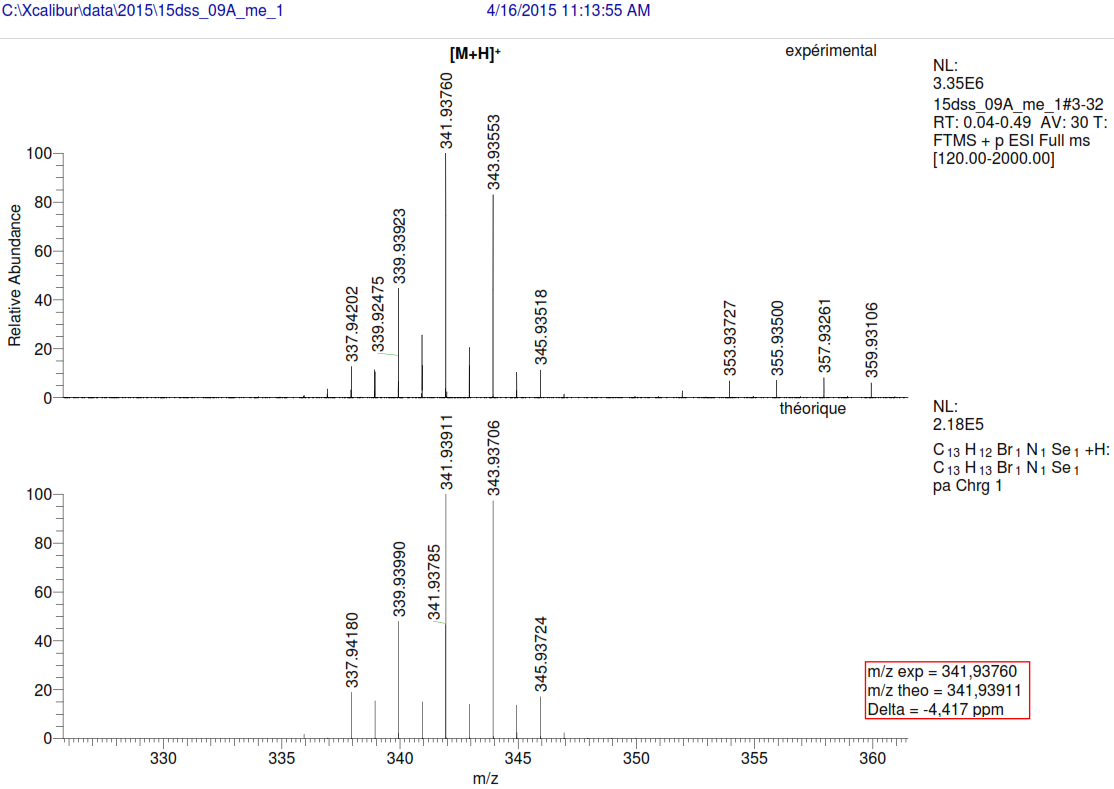


- 1. **4-((4-((4-Bromobenzyl)selanyl)phenyl)amino)-4-oxobut-2-enoic acid (3)**

A solution of 4-((4-bromobenzyl)selanyl)aniline (**2**) (344 mg, 1 mmol) and maleic anhydride (98 mg, 1mmol). in dry toluene (5 mL) was stirred at r.t. for 3h. Its formation was monitored by TLC chloroform: methanol = 8:1, R_f_ = 0.36, puriﬁed by column silica gel chromatography with chloroform: methanol = 6:1. Yellow solid; Yield: 412.66 mg (94%); mp 215-217 ^o^C. ^1^H NMR (300 MHz, DMSO) δ 10.45 (s, 1H, COOH), 7.55 (d, *J* = 8.6 Hz, 2H, Ar-H), 7.46 – 7.37 (m, 4H, Ar-H), 7.17 (d, *J* = 8.4 Hz, 2H, Ar-H), 6.45 (d, *J* = 12.1 Hz, 1H, =CH), 6.30 (d, *J* = 12.0 Hz, 1H, CH=), 4.04 (s, 2H, SeCH_2_); ^13^C NMR (75 MHz, DMSO) δ 167.31, 163.71, 139.14, 138.42, 134.02, 132.06, 131.60, 131.35, 130.90, 124.14, 120.54, 120.15, 30.71; MS (ESI): *m/z* = found 439.97 [M^+^+1]; calcd. 438.93 [M^+^]; HRMS calcd. for C_17_H_14_BrNO_3_Se [M^+^+Na]: 461.92145, found 461.92022 [M^+^+Na].

**
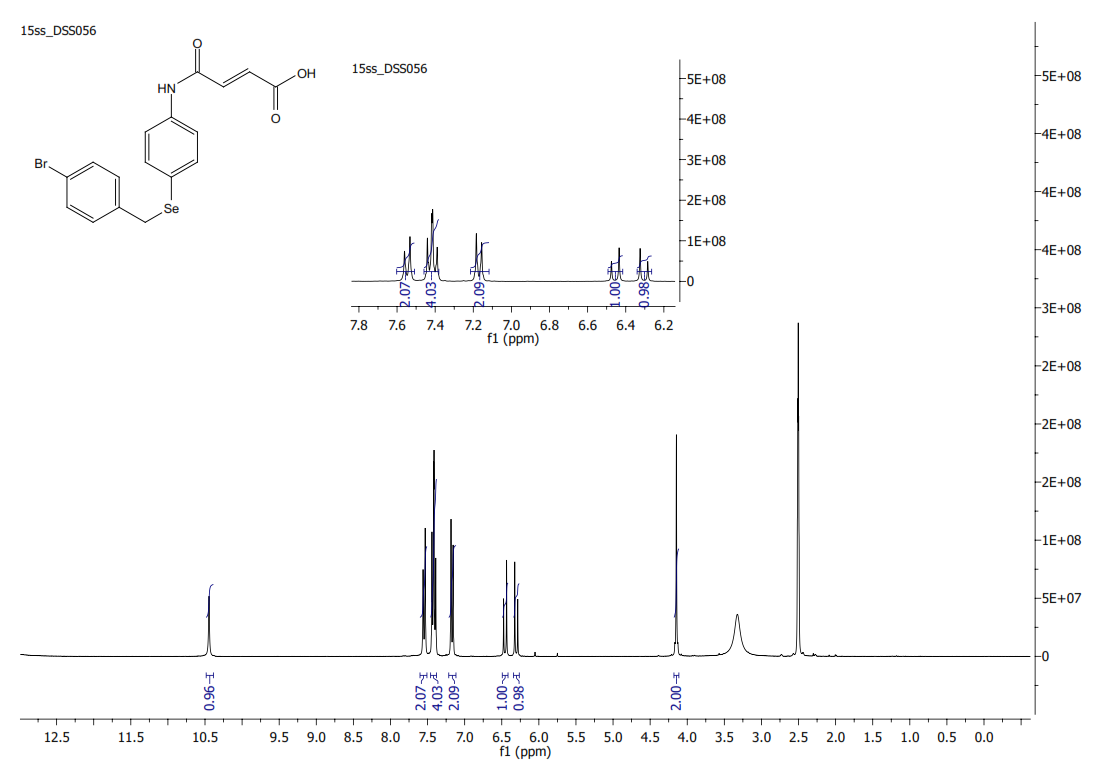
**

**
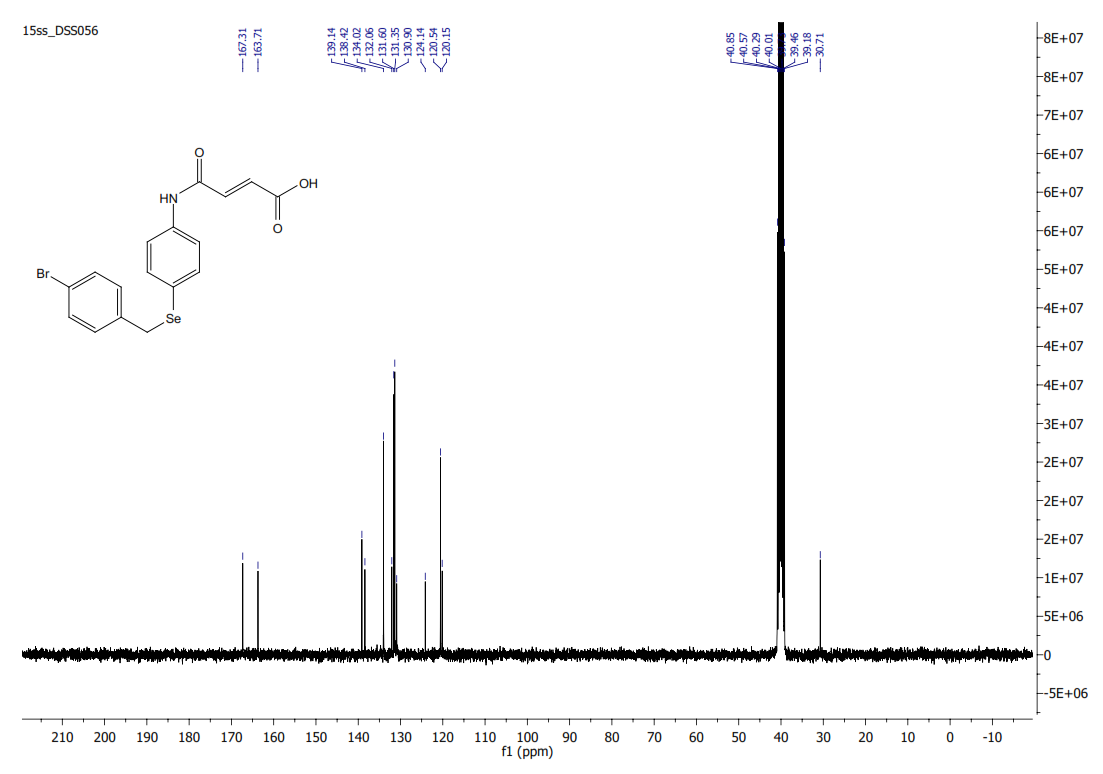
**

**
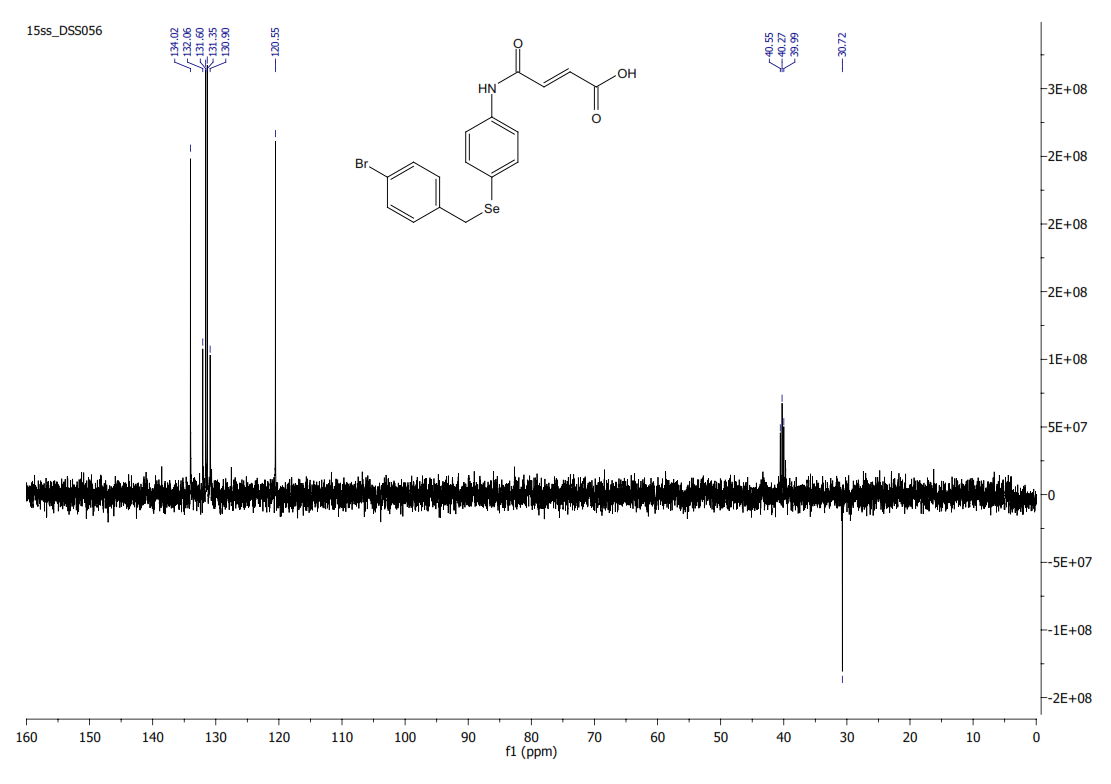
**


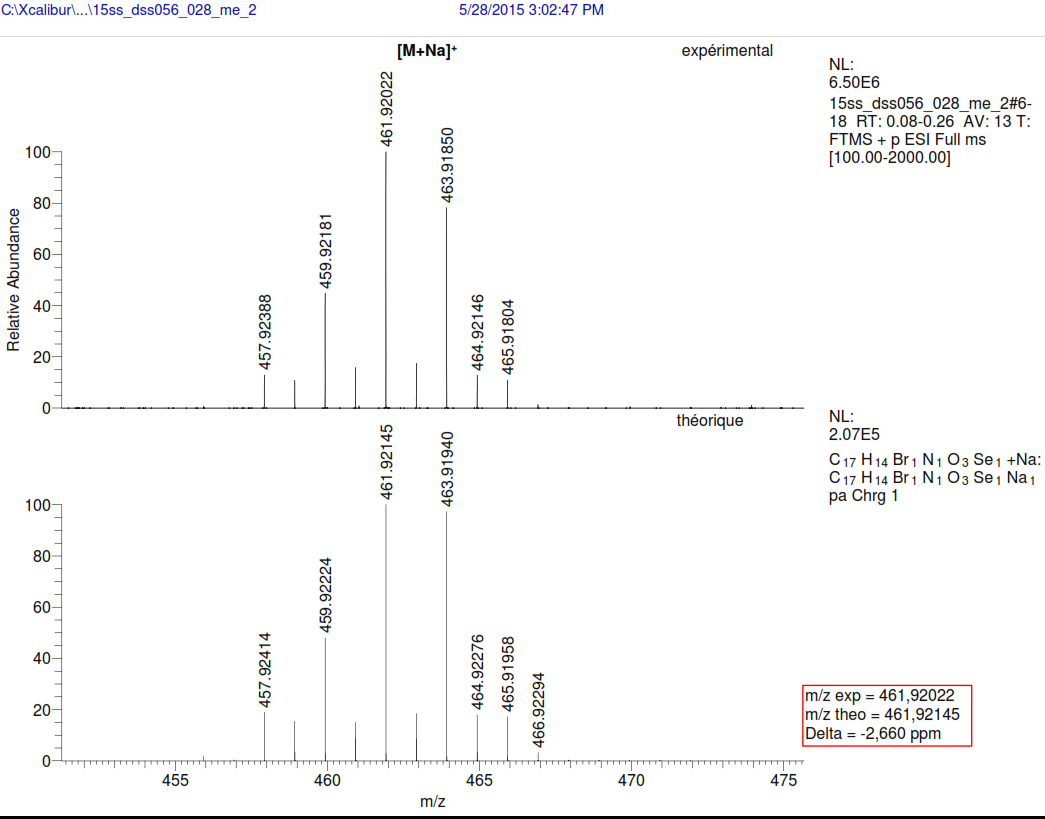


- 1. **5-((4-((4-Bromobenzyl)selanyl)phenyl)imino)-1H-pyrrol-2(5H)-one (4)**

To compound **3** (439 mg, 1 mmol), acetic anhydride (3 ml) and 100 mg of sodium acetate was added and the mixture was gently heated for 2h at 50-60 ^°^C. Ice was added and the resulting mixture was extracted with CH_2_Cl_2_ (200 ml), dried with Mg_2_SO_4_. CH_2_Cl_2_ was evaporated and the residue was purified by silica gel chromatography. The progress of the product formation was followed by TLC petroleum ether: EtOAc = 8:1, R_f_ = 0.45, puriﬁed by column silica gel chromatography with petroleum ether: EtOAc= 6:1. Yellow solid; Yield: 324.17 mg (77%), mp 185-187 ^o^C. ^1^H NMR (300 MHz, CDCl_3_) δ 7.47 – 7.29 (m, 4H, Ar-H), 7.25 – 7.14 (m, 2H, Ar-H), 7.04 – 6.96 (m, 2H, Ar-H), 6.76 (d, *J* = 5.5 Hz, 1H, CH=), 6.62 (d, *J* = 5.5 Hz, 1H, CH=), 4.01 (s, 2H, SeCH_2_); ^13^C NMR (75 MHz, CDCl_3_) δ 166.94, 150.34, 143.16, 142.77, 137.50, 134.29, 134.02, 131.59, 130.49, 127.93, 125.94, 120.83, 31.52; MS (ESI): *m/z* = found 474.92 [M^+^+CH_3_OH+Na]; calcd. 420.92 [M^+^]; HRMS calcd. for C_17_H_12_BrNO_2_Se [M^+^+1]: 475.93688, found 475.93544 [M^+^+CH_3_OH+Na].

**
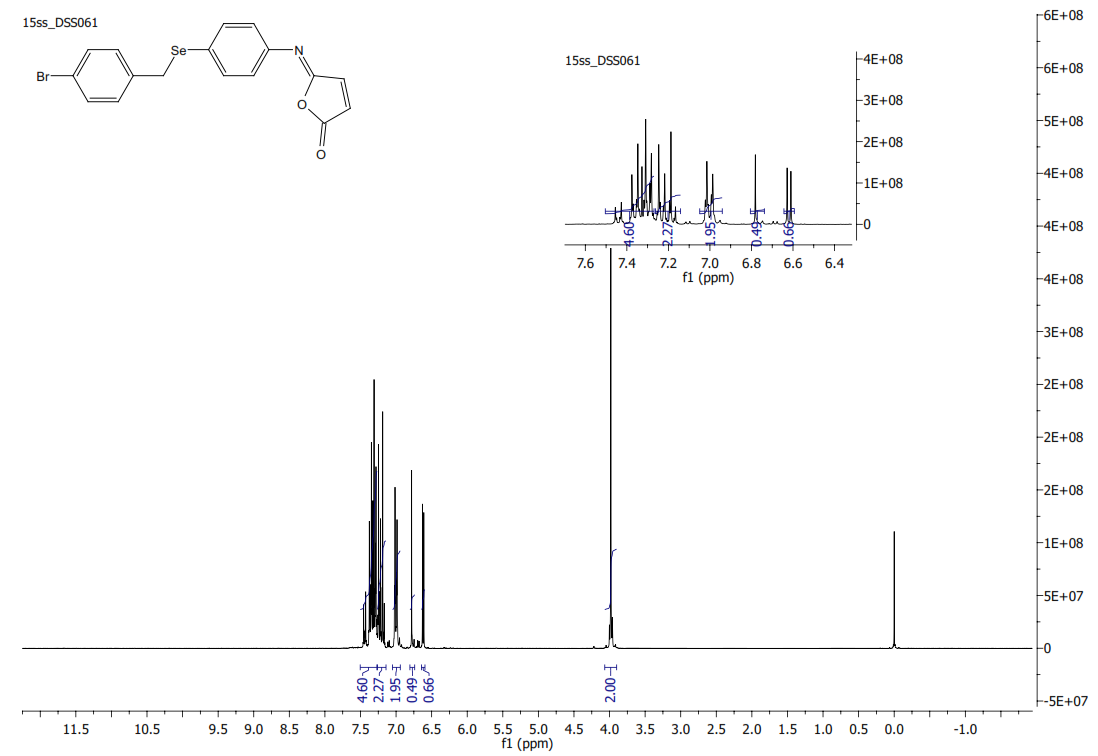
**


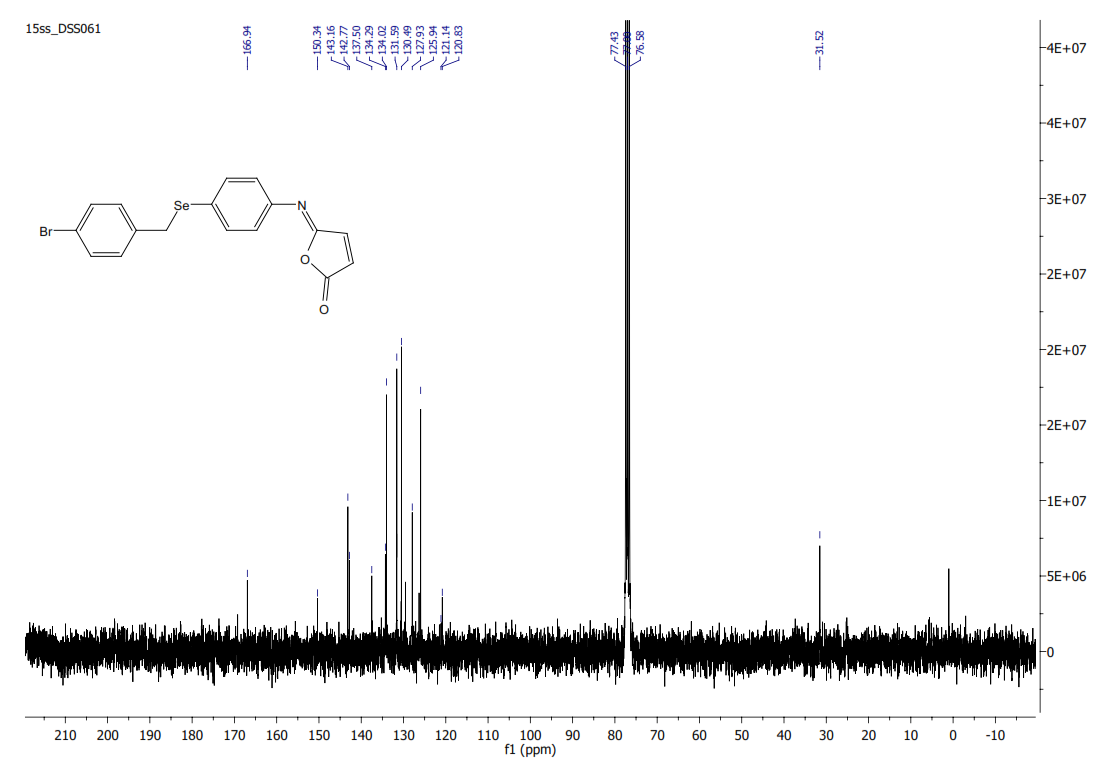


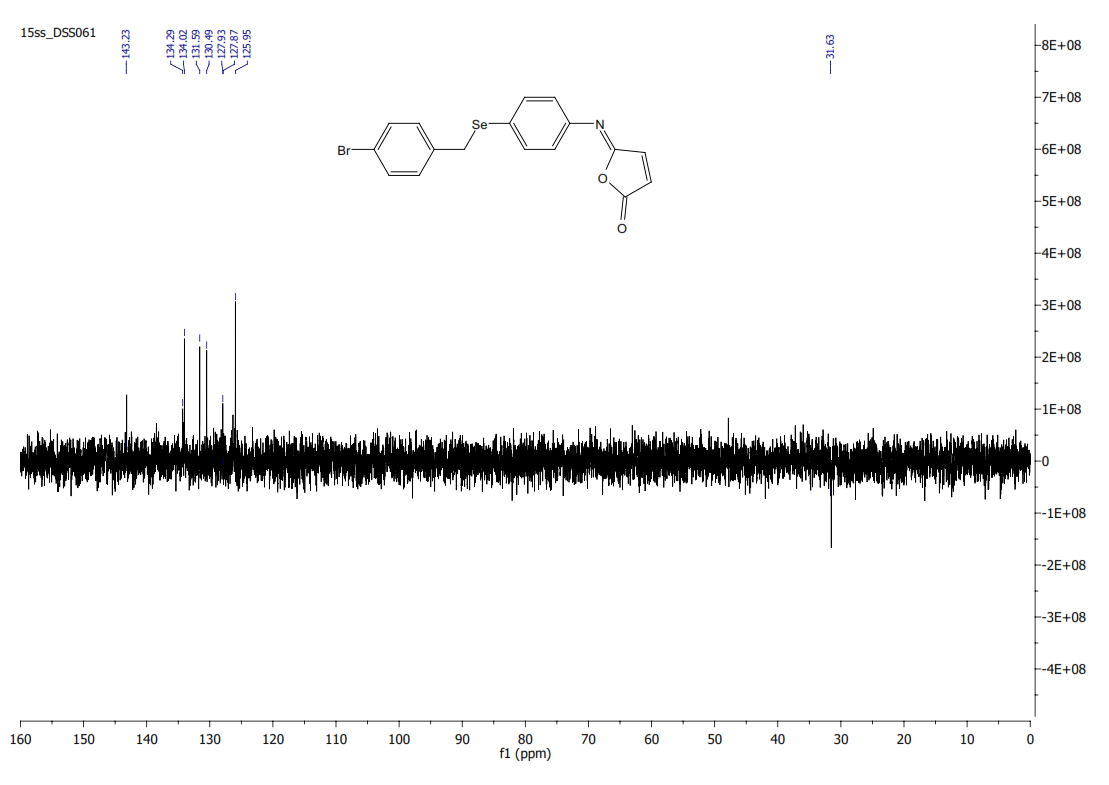


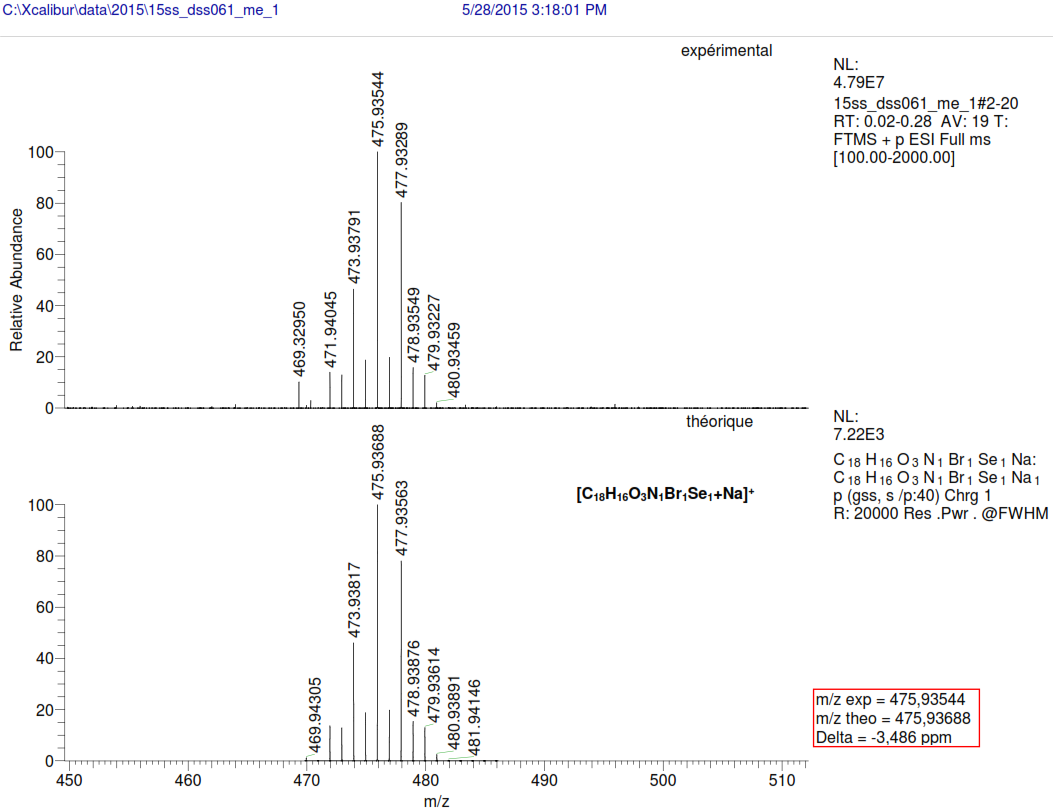


1. Shaaban, S.; Vervandier-Fasseur, D.; Andreoletti, P.; Zarrouk, A.; Richard, P.; Negm, A.; Manolikakes, G.; Jacob, C.; Cherkaoui-Malki, M. Cytoprotective and antioxidant properties of organic selenides for the myelin-forming cells, oligodendrocytes. *Bioorg Chem* **2018**, *80*, 43-56, doi:10.1016/j.bioorg.2018.05.019.

2. Shaaban, S.; Zarrouk, A.; Vervandier-Fasseur, D.; Al-Faiyz, Y.S.; El-Sawy, H.; Althagafi, I.; Andreoletti, P.; Cherkaoui-Malki, M. Cytoprotective organoselenium compounds for oligodendrocytes. *Arabian Journal of Chemistry* **2021**, *14*, 103051.

3. El-Senduny, F.F.; Shabana, S.M.; Rösel, D.; Brabek, J.; Althagafi, I.; Angeloni, G.; Manolikakes, G.; Shaaban, S. Urea-functionalized organoselenium compounds as promising anti-HepG2 and apoptosis-inducing agents. *Future Medicinal Chemistry* **2021**, *13*, 1655-1677.

4. Shaaban, S.; Shabana, S.M.; Al-Faiyz, Y.S.; Manolikakes, G.; El-Senduny, F.F. Enhancing the chemosensitivity of HepG2 cells towards cisplatin by organoselenium pseudopeptides. *Bioorg Chem* **2021**, *109*, 104713, doi:10.1016/j.bioorg.2021.104713.

5. Shaaban, S.; Zarrouk, A.; Vervandier-Fasseur, D.; S.Al-Faiyz, Y.; El-Sawy, H.; Althagafi, I.; Andreoletti, P.; Cherkaoui-Malki, M. Cytoprotective organoselenium compounds for oligodendrocytes. *Arabian Journal of Chemistry* **2021**, *14*, doi:10.1016/j.arabjc.2021.103051.
